# Noncovalent Assembly and Catalytic Activity of Hybrid Materials Based on Pd Complexes Adsorbed on Multiwalled Carbon Nanotubes, Graphene, and Graphene Nanoplatelets

**DOI:** 10.1021/acs.inorgchem.2c01559

**Published:** 2022-08-04

**Authors:** Alba M. Valbuena-Rus, Matteo Savastano, Paloma Arranz-Mascarós, Carla Bazzicalupi, María P. Clares, María L. Godino-Salido, María D. Gutiérrez-Valero, Mario Inclán, Antonio Bianchi, Enrique García-España, Rafael López-Garzón

**Affiliations:** † Department of Inorganic and Organic Chemistry, University of Jaén, 23071 Jaen, Spain; ‡ Department of Chemistry “Ugo Schiff”, University of Florence, Via della Lastruccia 3-13, 50019 Sesto Fiorentino, Italy; § National Interuniversity Consortium of Materials Science and Technology (INSTM), Via G. Giusti 9, 50121 Florence, Italy; ∥ ICMol, Department of Inorganic Chemistry, University of Valencia, C/Catedrático José Beltrán 2, 46980 Paterna, Spain

## Abstract

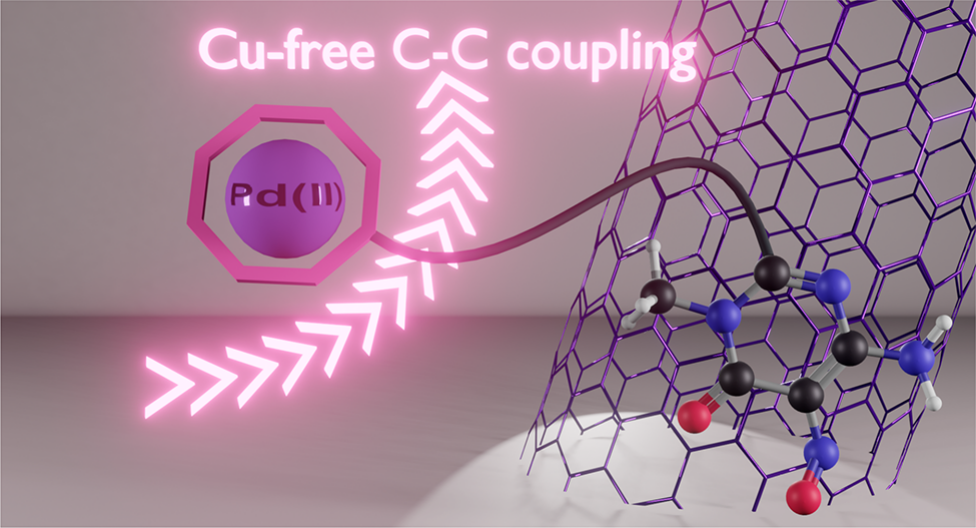

Green catalysts with excellent performance in Cu-free Sonogashira coupling reactions can be prepared by the supramolecular decoration of graphene surfaces with Pd(II) complexes. Here we report the synthesis, characterization, and catalytic properties of new catalysts obtained by the surface decoration of multiwalled carbon nanotubes (MWCNTs), graphene (G), and graphene nanoplatelets (GNPTs) with Pd(II) complexes of tetraaza-macrocyclic ligands bearing one or two anchor functionalities. The decoration of these carbon surfaces takes place under environmentally friendly conditions (water, room temperature, aerobic) in two steps: (i) π–π stacking attachment of the ligand via electron-poor anchor group 6-amino-3,4-dihydro-3-methyl-5-nitroso-4-oxo-pyrimidine and (ii) Pd(II) coordination from PdCl_4_^2–^. Ligands are more efficiently adsorbed on the flat surfaces of G and GNPTs than on the curved surfaces of MWCNTs. All catalysts work very efficiently under mild conditions (50 °C, aerobic, 7 h), giving a similar high yield (90% or greater) in the coupling of iodobenzene with phenylacetylene to form diphenylacetylene in one catalytic cycle, but catalysts based on G and GNPTs (especially on GNPTs) provide greater catalytic efficiency in reuse (four cycles). The study also revealed that the active centers of the ligand-Pd type decorating the support surfaces are much more efficient than the Pd(0) and PdCl_4_^2–^ centers sharing the same surfaces. All of the results allow a better understanding of the structural factors to be controlled in order to obtain an optimal efficiency from similar catalysts based on graphene supports.

## Abstract 2

Green catalysts with high efficiency in the Cu-free Sonogashira C−C coupling reactions can be prepared by the supramolecular functionalization of carbon materials.

## Introduction

A sustainable economy, which is one of the most compelling demands of the moment, requires the achievement of two main objectives: climate neutrality, which implies energy saving and the production of energy from renewable sources, and production cycles which, in addition to being energy-saving, produce minimal waste materials. Catalysts can be of great help in this regard;^1−9^ among them, those based on both metal ion complexes and metal nanoparticles supported on graphene-like surfaces are gaining significant importance in facing the challenge (Table S1).^10−20^

As for the synthesis of materials, the use of catalysts is often essential for the correct course of reactions and to obtain acceptable quantities of products with reasonable energy consumption, in line with the objectives of energy savings and waste reduction. For example, the Sonogashira cross-coupling reaction, which leads to the formation of a C–C bond between an sp-hybridized terminal C atom of an alkyne and an sp^2^ C atom of an aryl or vinyl halide, has been widely used for the synthesis of a large variety of compounds. This method was first described in 1975 as a reaction that occurs easily in the presence of PdCl_2_(PPh_3_)_2_ as a catalyst, aided by Cu(I) as a cocatalyst.^21^ Copper was subsequently eliminated when Pd alone proved sufficient if properly coordinated, thus eliminating an additional compound and partially remediating the inconvenience of working under a protected atmosphere. As a result, many new Pd catalysts were developed, of both homogeneous and heterogeneous nature,^22^ and alternative metals were also explored.^23−25^

Heterogeneous catalysts appear to be of more practical use because they can be easily recovered from the reaction environments and can also be reused if their stability allows. Various solid supports have been proposed for their assembly, including many carbon-based materials.^10−13,26−33^ Pd-based micellar catalysts have also been shown to be very effective, making Sonogashira coupling reactions possible in water with good yields and with sustainable loadings of the precious metal.^34,35^

**Scheme 1:**
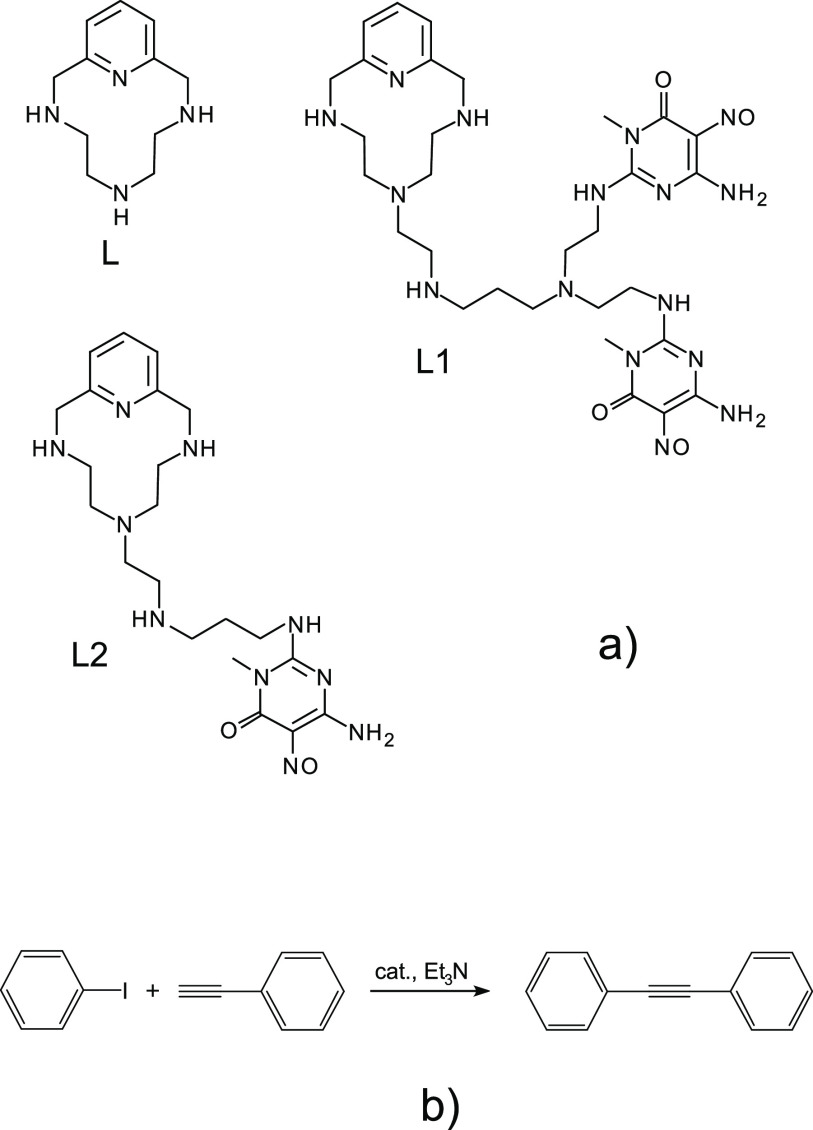
(a) Ligands and (b) the Sonogashira Reaction Studied in This Work

We have recently reported the catalytic behavior of two hybrid materials, G-L1-Pd and MWCNT-L2-Pd, in the copper-free Sonogashira reaction of iodobenzene (IB) with phenylacetylene (PA) to obtain diphenylacetylene (C–C bond formation).^36,37^ The catalysts were obtained by a two-step procedure. First, the ligands, L1 and L2 (Scheme 1), were adsorbed on commercial graphene (G) and multiwalled carbon nanotubes (MWCNTs), respectively, yielding G-L1 and MWCNT-L2 hybrids. Then, the adsorption of PdCl_4_^2–^ on these hybrids was carried out until a 1:1 ligand/Pd molar ratio was obtained, thus providing the catalysts G-L1-Pd and MWCNT-L2-Pd in which most of the Pd(II) was adsorbed via complexation by the common macrocyclic polyamine function of the corresponding ligand. The adsorption of L1 and L2 on the carbon substrates was ensured by the π–π stacking interaction of the 6-amino-3,4-dihydro-3-methyl-5-nitroso-4-oxo-pyrimidine ligand moieties (two in L1, one in L2) with the graphene surfaces of MWCNTs and G. The use of this adhesive pyrimidine residue was initially introduced by some of us for the functionalization of activated carbon (AC) aimed at the removal of chromate anions from aqueous solutions^38^ and was later extended to the generation of new materials for further remediation purposes (cation and anion sequestration)^39−45^ as well as for applications in fuel cells (oxygen reduction reactions),^46−50^ hydrogenation processes,^51^ and the photochemical generation of hydrogen.^52^

**Figure 1:**
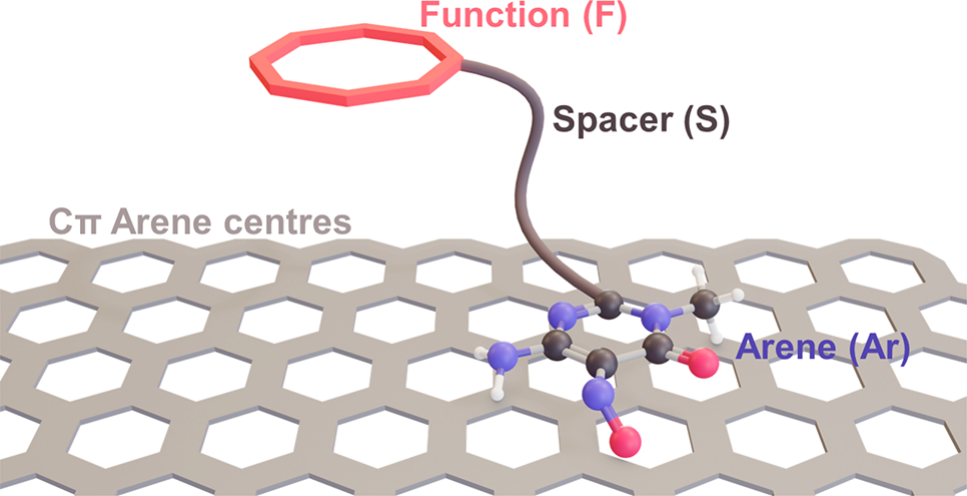
Schematic representation of the arene-spacer-function (Ar-S-F) ligands and their interaction with the Cπ arene centers of substrates.

Striking features of this mild supramolecular functionalization using Ar-S-F-type ligands (Figure 1) are the highly irreversible character of ligand adsorption on graphene surfaces via the Ar function, the achievement of homogeneous coverage, the preservation of the electronic properties of the substrate, and the ease of changing the coordinating F function (targeted ligand design).^53^

Catalysts G-L1-Pd and MWCNT-L2-Pd showed enhanced activity in the above-mentioned Sonogashira reaction, providing high yields, reusability, and relatively short reactions times. For instance, in the presence of MWCNT-L2-Pd, yields greater than 90% were obtained in a short time (2 h) while working under green conditions (water, 50 °C, aerobic atmosphere).^36^ Excellent conversion yields (90%) were also obtained in the presence of G-L1-Pd under the same experimental conditions but with a somewhat longer reaction time (14 h).^37^

These studies also demonstrated that the good performances of these catalysts are closely related, among other factors, to the stability of the Pd(II)-polyamine complexes and to the stable interaction of these complexes with the graphene surfaces.

During their reuse, however, these catalysts undergo some loss of activity. In the case of MWCNT-L2-Pd, this is due to the progressive desorption of L2-Pd, which results in the loss of active catalytic centers.^36^ In reaction to this, we decided to strengthen the interaction of the complex with the graphene surface by preparing a ligand with two pyrimidine anchor groups (L1) which was fixed on the flat surface of G to give G-L1-Pd.^37^ Indeed, the stronger interaction preserves G-L1-Pd from the desorption of L1-Pd. However, a loss of activity greater than for MWCNT-L2-Pd was observed during the catalyst reuse, which this time occurs parallel to the progressive agglomeration of the Pd(0) nanoparticles formed in the first reaction cycle. This phenomenon, not observed in the case of MWCNT-L2-Pd, was ascribed to some interaction of the pyridine group of the macrocyclic pendant with the surface of G, which probably weakens the stability of the L1-Pd complex.^37^

In brief, MWCNT-L2-Pd is better performing than G-L1-Pd but is affected by the desorption of L2-Pd, while the latter does not have this drawback but is poorer performing than the former and suffers from the reduction of Pd(II) to Pd(0). Accordingly, one might expect that better catalysts can be obtained by exchanging substrates and ligands (complexes) (i.e., by preparing G-L2-Pd and MWCNT-L1-Pd).

To explore this possibility, we have prepared the hybrid materials MWCNT-L1-Pd and G-L2-Pd and studied their catalytic activity in four reaction cycles of the above-cited copper-free Sonogashira reaction. In this case, the preparation was carried out under suitable conditions so that the final catalysts contain a molar excess of Pd relative to L1 and L2 ligands with the purpose of gaining more insight into the catalytic activity of different Pd centers, both coordinated to the ligand functions and directly attached to the support surfaces. Indeed, with regard to the latter, in previous studies we found that even hybrids obtained by the direct adsorption of PdCl_4_^2–^ on both MWCNT and G exhibit single-use non-negligible catalytic activity.^36,37^

Furthermore, taking into account the behavior observed for G-L2-Pd, it was considered opportune to extend the above study to the analogous Pd catalysts obtained by adsorbing L1 and L2 on graphene nanoplatelets (GNPT).

## Experimental Section

### Materials

Few-layered graphene (no. 2191YJ) was provided by NanoAmor (USA). According to the manufacturer, the product has sheet lateral sizes of between 2 and 10 μm and one to three layers. Before use, it was suspended in water under stirring for 24 h to remove the labile oxygen groups and then separated by filtration and air-dried. Characterization data of the obtained material, G, were published previously.^52,54,55^

Commercial multiwalled carbon nanotubes (MWCNTs) were purchased from NanoAmor (USA). According to the data from the supplier, the product has an outside diameter of 8–15 nm, an inside diameter of 3–5 nm, and lengths of between 10 and 50 μm. The material was washed with water and air-dried before use in the experiments described below.

Graphene nanoplatelets (GNPTs) were supplied by Nanografi (Ankara, Turkey). The sample used contains C (92.2% by weight) and O (7.8% by weight) as determined from XPS spectra (Figure S1). According to the data provided by the manufacturer, the size of the sheets of this material is 3 nm. The material was washed with water and air-dried, as described above for G and MWCNTs, before use.

Ligands L,^56^ L1,^37^ and L2^36^ were prepared as previously described. All solvents and other chemicals were of analytical grade and used without any further purification.

Crystals of [Pd(HL)Br_2_]Cl_0.74_Br_0.26_·H_2_O (**2**) suitable for XRD analysis were obtained by slow evaporation at room temperature of a solution obtained by adding, in small portions, 14.8 mg (0.0453 mmol) of K_2_PdCl_4_ dissolved in 3 cm^3^ of water to a boiling solution of L·3HBr (20.3 mg, 0.0452 mmol) dissolved in 4 cm^3^ of water. Orange crystals started growing after several days. When the solution had reduced to about 3 cm^3^, the crystals were filtered and air-dried. Yield: 72%. Elemental analysis: calcd. (%) for C_11_H_20_N_4_OPdCl_0.74_Br_2.26_: C, 24.58; H, 3.75; N, 10.42. Found: C, 24.42; H, 3.83; N, 10.35. The homogeneity of the sample was verified by comparing experimental and calculated X-ray powder diffraction spectra (Figure S2).

After the filtration of the crystals, the mother liquor was left to evaporate further. The solution evaporated to dryness, leaving an amorphous solid in which very few orange crystals were found. One of them was suitable for XRD analysis, which allowed the determination of its composition as [Pd(HL)Br_2_]Br (**1**).

### Preparation of the Catalysts

The catalysts were prepared by a two-step procedure. In the first step, ligands L1 and L2 were adsorbed on MWCNTs, G, and GNPTs to form MWCNT-L1, G-L2, GNPT-L1, and GNPT-L2 carbon support-ligand hybrids; in the second steps, they were used to prepare carbon support-ligand-Pd materials by the adsorption of PdCl_4_^2–^.

### Preparation of the Carbon Support-Ligand Hybrids

The carbon support-ligand hybrids were prepared through adsorption experiments on G, MWCNTs, and GNPTs of aqueous solutions of ligands L1 and L2. The experimental conditions for the adsorption experiments were selected, by preliminary tests in water, in order to obtain the maximum load of the corresponding ligand on each adsorbent. In the experiments, 0.100 g of the carbon support was suspended in 400 cm^3^ of a 7.5 × 10^–4^ M aqueous ligand solution. The pH of the ligand solutions was adjusted to 5.0 by adding suitable amounts of a 0.1 M HCl solution. The ligand/carbon adsorbent mixtures, contained in a plastic flask, were stirred in an incubator shaker (CertomatTM IS SARTORIUS), thermostated at 298.1 K, until adsorption equilibrium was reached (4 to 6 days), after which the corresponding solid phases were separated by filtration, repeatedly washed with distilled water, and air-dried. The amounts of ligand adsorbed were determined as the difference in absorbance at 301 nm, in the case of L1, and at 304 nm, in the case of L2, between the initial and the equilibrated solutions. The resulting hybrids containing 0.21, 0.45, 0.55, and 0.82 mmol of ligand per gram of carbon support were labeled as MWCNT-L1, G-L2, GNPT-L1, and GNPT-L2. These ligand loads were consistent with those obtained from the atomic composition of the solids calculated from survey XPS spectra.

### Preparation of Carbon Supports-Ligand-Pd(II) Materials

The carbon supports-ligand-Pd(II) materials were prepared by the adsorption of a K_2_PdCl_4_ solution on the appropriate carbon support-ligand hybrid previously obtained. Accordingly, 0.100 g of the hybrid was mixed in a plastic flask with 400 mL of a 1 M KCl aqueous solution containing 7.5 × 10^–4^ M K_2_PdCl_4_, whose pH was adjusted to 5.0 by the addition of aqueous HCl. This pH was selected as the best compromise between minimizing proton competition with Pd^2+^ coordination to the ligand amino groups and preventing the formation of Pd^2+^ hydroxy species that, at this pH, are not yet formed (i.e., all the Pd^2+^ is present as [PdCl_4_]^2–^).^53^ The suspensions were shaken in an incubator shaker and thermostated at 298.1 K for 3 days until adsorption equilibrium was reached (i.e., until the UV absorbance of the Pd^2+^ solution at λ = 474 nm remained constant over time). Then the amounts of adsorbed Pd in the resulting materials MWCNT-L1-Pd, G-L2-Pd, GNPT-L1-, and GNPT-L2-Pd (0.64, 0.92, 1.03, and 1.59 mmol of Pd per gram of hybrid, respectively) were determined from the atomic composition obtained from the corresponding XPS spectra of the solids.

### Characterization of the Obtained Solids

The X-ray photoelectron spectroscopy (XPS) spectra of the solids were obtained in a Kratos Axis Ultra DLD spectrometer. Monochromatic Al/Mg Kα radiation in constant analyzer energy mode with pass energies of 160 and 20 eV (for the survey and high-resolution spectra, respectively) was used. The C 1s transition at 284.8 eV was used as a reference to obtain the heteroatom binding energies. The accuracy of the binding energy (BE) values was ±0.2 eV.

Survey XPS spectra were used to obtain data on the elemental composition of solid materials. The chemical nature of the elemental components of the samples was determined from high-resolution XPS spectra. These data provide structural information on the prepared solids and on the possible structural changes suffered by the catalysts when reused in successive reaction steps of the Sonogashira reaction studied between phenylacetylene and iodobenzene.

Transmission electron microscopy (TEM) images, high-resolution transmission electron microscopy (HRTEM) images, and mapping measurements were collected on a HAADF FEI TITAN G2 microscope, with resolutions of 0.8 Å in TEM mode and 1.4 Å in STEM mode. The microscope was operated with a working tension of 300 kV. The counting of Pd(0) nanoparticles in the solids was carried out with digital electron micrographs with the ImageJ program (free software, Wayne Rasband, USA).

The textural characteristics of GNPT and GNPT-L1 were obtained by nitrogen adsorption at 77 K by using ASAP 2020 equipment (Figure S3). The surface areas were obtained by applying the Brunauer–Emmett–Teller (BET) equation to the adsorption data, and the pore volumes were determined by the Barret–Joyner–Halende (BJH) method (Table S2).^57^

### General Procedure for the Sonogashira Reaction

A mixture of iodobenzene (1 mmol), phenylacetylene (1.5 mmol), Et_3_N (2 mmol), H_2_O (1 cm^3^), and the catalysts (from 15 to 25 mg), with reactants/Pd(II) = 100, was stirred under aerobic conditions at a constant temperature (50 °C). The progress of the reaction was monitored by gas chromatography (GC). After completion, CHCl_3_ (10 mL) was added to the reaction mixture and the catalyst was recovered by filtration and washed with CHCl_3_ and H_2_O. The organic layers were collected and dried over anhydrous Na_2_SO_4_. The analysis of the reaction products in the organic phase was performed by GC using a 7820A Agilent GC System chromatograph with an Agilent 190915-433 column (30 m × 250 μm × 25 μm) and a flame ionization detector (FID). A further check of the reaction products was carried out by random sampling to analyze the composition of the reaction crudes by means of ^13^C and ^1^H NMR spectroscopy. For illustrative purposes, some of the results obtained are shown in Figure S4 and Table S3. The corresponding NMR spectra were obtained by using a 400 MHz NMR spectrometer (Bruker, AVANCE NEO 4400). The procedure was as follow: the crude products were evaporated under reduced pressure, and then 30 mg of each residue was dissolved in 0.5 mL of CDCl_3_ and the NMR spectra were recorded. The recovered catalysts were reused for three additional runs, repeating the same procedure.

### Crystal Structure Determination

Orange crystals of [Pd(HL)Br_2_]Br (**1**) and [Pd(HL)Br_2_]Cl_0.74_Br_0.26_·H_2_O (**2**) were used for X-ray diffraction analysis. A summary of the crystallographic data is reported in Table S4. The integrated intensities were corrected for Lorentz and polarization effects, and an empirical absorption correction was applied.^58^ Crystal structures of **1** and **2** were solved by using SHELXS-97,^59^ and refinements were performed by means of full-matrix least-squares using SHELXL version 2014/7.^60^ Non-hydrogen atoms were anisotropically refined. Hydrogen atoms were introduced as riding atoms with thermal parameters calculated in agreement with the linked atom. The water hydrogens in **2** were not localized in the ΔF map and were not introduced in the calculation. Compound **2** was found to be a solid solution of 74% [Pd(HL)Br_2_]Cl·H_2_O and 26% [Pd(HL)Br_2_]Br·H_2_O. Bromide and chloride anions share the same position. They were freely refined, with sum of their occupancy factors fixed to 1.

## Results and Discussion

### Formation of Pd(II) Complexes

L1 and L2 contain the common macrocyclic unit 3,6,9-triaza-1-(2,6)-pyridinacyclodecaphane (L in Scheme 1), which was shown to be the coordination site for Pd(II) ions.^36,37^ Complexation of Pd(II) by the two ligands is a very slow process. Nevertheless, once the complexes are formed, protonation equilibria in which they are involved (not occurring on the macrocyclic coordination site) are fast in solutions with pH > 2.5 and can be studied by potentiometric titrations. In more acidic media, the equilibration of titrated solutions becomes very slow, probably because of the competition between ligand protonation (protonation of macrocycle amine groups) and Pd(II) complexation processes. According to equilibrium data and other analytical results obtained in chloride-rich media, the main species formed above pH 2.5 contain Pd(II) ions coordinated to three nitrogen atoms of the macrocyclic unit and one chloride anion.^36,37^ This coordination environment is retained after the adsorption on MWCNTs, G, and GNPts, according to previous^36,37^ and present studies.

Attempts to obtain crystals of the Pd(II) complexes with L1 and L2 suitable for XRD analysis were unsuccessful, preventing the visualization of their structures. Nevertheless, we have now managed to prepare crystals of the Pd(II) complex with the macrocyclic ligand L, the coordination unit of L1 and L2, and to resolve their structures. Two samples of crystals corresponding to [Pd(HL)Br_2_]Br (**1**) and [Pd(HL)Br_2_]Cl_0.74_Br_0.26_·H_2_O (**2**) were obtained from acidic solutions (Experimental Section).

**Figure 2:**
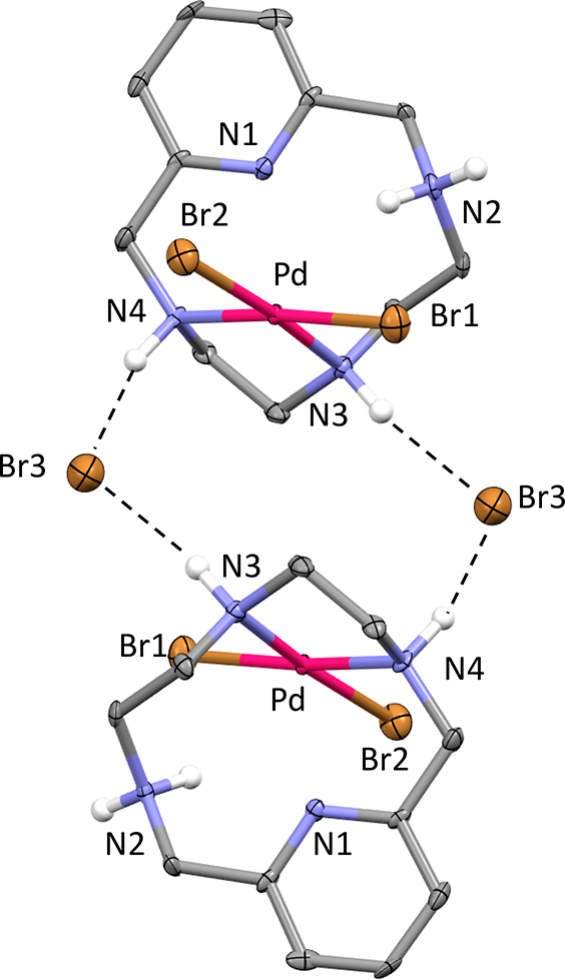
Centrosymmetric dimer of [Pd(HL)Br_2_]^+^ cations in [Pd(HL)Br_2_]^+^Br (**1**) H-bonded to the Br^–^ counterions.

These compounds crystallize in the triclinic (**1**) and monoclinic (**2**) systems. Both contain the [Pd(HL)Br_2_]^+^ complex cation, as the bromide salt in **1** and as the monohydrated chloride/bromide solid solution (74% Cl^–^, 36% Br^–^) in **2**. Despite the different packing, the protonated complex cation assumes almost the same conformation (RMSD = 0.126 Å evaluated on all atoms), defining very similar dimeric motifs in **1** (Figure 2) and **2** (Figure S5). The macrocycle is slightly bent, with a dihedral angle between the aromatic and the aliphatic portions of the ligand of 156 and 149° in **1** and **2**, respectively. The Pd(II) is coordinated by two aliphatic N atoms, one of which is benzylic, and two Br^–^ anions, giving rise to the expected square-planar geometry. The second benzylic N atom is protonated while the pyridine one is not involved in metal binding. Pd–N and Pd–Br bond distances (Table S5) agree with the corresponding reference values evaluated by CSD statistics (Pd–N 2.05(6) Å, Pd–Br 2.47(7) Å). The coordination plane is almost perpendicular to the pyridine ring (82° in **1**, 89° in **2**). Two of these metal complexes are joined together to form dimeric motifs (Figure 2 and Figure S5), where the coordinated N atoms of each complex unit are H-bonded to bridging Br^–^ anions in **1** and bridging Cl^–^ or Br^–^ anions in solid solution **2** (N···Br and N···Cl H-bond distances in the range of 3.194(8)–3.23(2) Å, Table S6). The Pd–Pd intermetallic distance is 5.688(2) Å in **1** and 5.4293(6) Å in **2**, and the bridging anions are 6.670(4) Å apart from each other in **1** and 6.98(1) Å (Cl^–^) or 7.02(2) Å (Br^–^) apart from each other in **2**.

Complexation of Pd(II) by L is also a slow process in solution, similar to those observed for L1 and L2. The reorganization of the [Pd(HL)Br_2_]^+^ complex, occurring upon deprotonation, to allow coordination of an additional N atom in competition with an already coordinated Br^–^ anion would be the slow process accompanying the formation of [Pd(L)Br]Br from its protonated form in solution. According to the similarity in the coordination sites of L, L1, and L2, a similar process might be representative of Pd(II) coordination by all of them and may provide a possible representation of the structure of the complexes formed in acidic media.

Nevertheless, it is important to stress that this crystal structure is not necessarily a faithful representation of the structure of Pd(II) complexes with L1 and L2. This is even more stringent if we want to refer to the structures of the complexes formed by L1 and L2 in solution. The bulky substituents that transform L into L1 and L2 determine a different flexibility of the ligands which can affect their coordination to metal ions, especially in the case of Pd(II) having strict coordination requirements. Furthermore, these substituents modify the basicity and the donating properties of the amine groups with possible repercussions in which nitrogen atoms will be more inclined toward metal coordination or protonation.

Beyond any structural speculation, this crystal structure is evidence of the robustness of the complexes with Pd(II) coordinated to L units, being resistant to dissociation even in very competitive media (Cl^–^, Br^–^), including very acidic ones (pH 1) such as those used for the preparation of [Pd(HL)Br_2_]. This is a property of great importance for complexes to be used as catalysts because it allows their use even under severe conditions.

### Preparation and Characterization of MWCNT-L1-Pd and G-L2-Pd Hybrids

The catalysts were obtained through a two-step procedure similar to that described above for MWCNT-L2-Pd(II) and G-L1-Pd(II) (i.e., the adsorption of the corresponding ligand on the carbon support and then the adsorption of PdCl_4_^2–^ on the carbon support-ligand hybrid resulting from the first step).

The main constituents of the G used in this work (one to three sheets units) were C (94.8% by weight) and O (4.6% by weight), and the nature of the oxygen functions (carboxyl, carbonyl, hydroxyl, and epoxy groups) was reported in previous papers.^52,54^ As shown by XPS analysis, the main components of the MWCNTs used in this work are C (97.9% by weight) and oxygen (2.1% by weight); no other elements were detected in significant amounts (Figure S1). The nature of the oxygen functions (carboxyl, carbonyl, and phenol) was determined from the HR XPS spectrum in both the C 1s and O 1s ranges.

**Figure 3:**
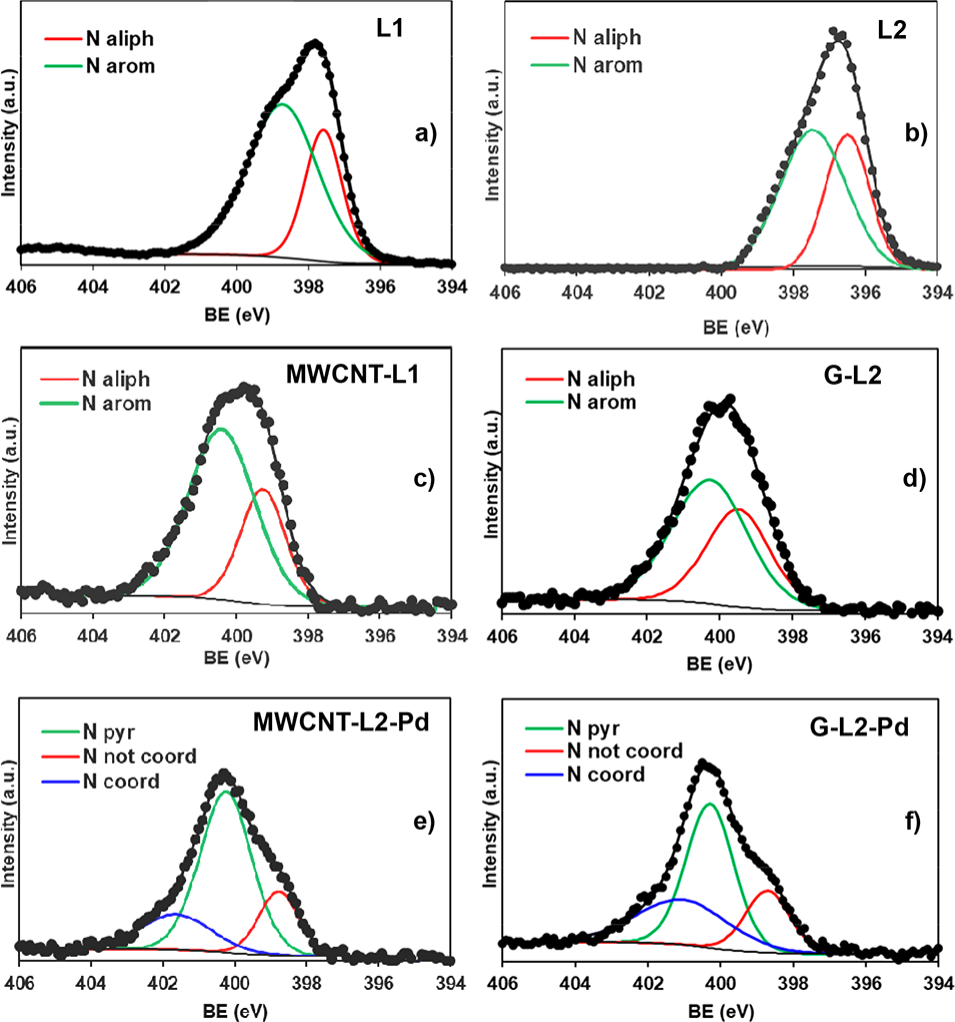
High-resolution XPS spectra in the N 1s region of (a) L1, (b) L2, (c) MWCNT-L1, (d) G-L2, (e) MWCNT-L2-Pd, and (f) G-L2-Pd.

Extensive studies on the adsorption of C(2)-substituted derivatives of 6-amino-3,4-dihydro-3-methyl-5-nitroso-4-oxo-pyrimidine on carbon substrates having common graphene surfaces (activated carbons,^38,41,42,53^ carbon nanotubes,^36^ and graphene^37,43,52^) indicated that ligand adsorption takes place through strong π–π interactions of the pyrimidine moiety of the ligand with the basic arene centers (Cπ) of the graphene surface. The significant strength of such interactions is due to van der Waals forces and an electrostatic component arising from the electronic properties of the pyrimidine moiety.^52^ This kind of interaction is recognized through the high-resolution XPS spectra of the carbon support-ligand hybrids since the binding energy (BE) values of N 1s and O 1s components of the free pyrimidine moiety bear significant shifts upon adsorption on the graphene surfaces. These shifts occur because the interaction generates repulsion between the adjacent π clouds, which results in a deshielding of the atomic constituents of the pyrimidine residue. The high-resolution XPS spectra of MWCNT-L1 and G-L2 in the N 1s ranges appear in Figure 3 along with those previously reported for free L1^37^ and L2^36^ ligands. N 1s signals of these free ligands contain two components, which are assigned to aliphatic nitrogen (the lower-energy one) and aromatic nitrogen (the higher-energy one), whose relative intensities correspond to those of their molecular structures (i.e., 5aliph/11arom for L1 and 4aliph/6arom for L2). As shown in Figure 3, the components of aromatic nitrogen of both ligands undergo significant shifts toward higher BE values upon adsorption, in agreement with the above considerations. The components of aliphatic N atoms also shift similarly as a result of some protonation of the aliphatic amino groups occurring during the preparation of both MWCNT-L1 and G-L2 hybrids in water at pH 5.^36,37^

Similar shifts toward higher BE values are also experienced by the O 1s signals of both ligands upon adsorption. The wide signals due to the oxygen groups of the carbon substrates (MWCNT and G) are in the 530–535 eV range, and those of the oxygen functions of the free ligands (the C(6) and C(5)–NO groups of the pyrimidine) are found at about 529 eV. This relatively low value is due to the polar nature of the pyrimidine moiety whose two oxygen groups bear a significant negative charge.^52^ Thus, the lack of 529 eV signals in the O 1s spectra of the MWCNT-L1 and G-L2 hybrids (Figure S6) can be explained by considering that, similar to the case of N-aromatic atoms, interaction with the carbon substrate causes them to shift up to 530–535 eV, to be included in the wide O 1s signal of the corresponding carbon substrates.

The above XPS data confirm that the adsorption of the L1 and L2 ligands on MWCNT and G substrates, respectively, takes place by plane-to-plane attachment of the 6-amino-3,4-dihydro-3-methyl-5-nitroso-4-oxo-pyrimidine residue of both ligands to the graphene surface of the substrates. This type of interaction is similar to that observed for the adsorption of L1 on G and of L2 on MWCNT^36,37^ as well as for many similar molecules containing the same pyrimidine moiety when adsorbed on ACs, MWCNTs, and G, as previously described in detail. In most cases, this type of interaction results in an excellent preservation of Brönsted acid/base and coordination properties of the free functions attached to C(2)pyrim.^36,38,41−43,54^ However, depending on the characteristics and stereochemistry of the pendant function at C(2)pyrim and the pore structure of the carbon substrate, the properties of these free functions can be affected by adsorption. For example, when ACs are used as a substrate, the ligand molecules are adsorbed into very narrow pores and both stability and metal coordination abilities of the substituents on C(2)pyrim decay abruptly because of stereochemical restrictions.^61^ In the case of L1 and L2, the pyridine of the macrocyclic pendant is also likely to give some interaction with the graphene surfaces of MWCNT and G, in addition to the strong interaction of the pyrimidine group, thus hampering to some extent the protonation and metal ion complexation capacity of the macrocyclic unit.^36,37^

When the hybrid materials of the above types contain Ar-S-F ligands (Figure 1) with suitable metal complexation functions (F), the adsorption of metal ions occurs mainly through coordination to these functions.^36,37,43,52−54^

The preparation of MWCNT-L1-Pd and G-L2-Pd was carried out by adsorption experiments using a 5 × 10^–4^ M PdCl_4_^2–^ aqueous solution in the presence of MWCNT-L1 (L1, 0.21 mmol g^–1^) and G-L2 (L2, 0.45 mmol g^–1^), respectively (Experimental Section). The resulting materials MWCNT-L1-Pd and G-L2-Pd contained 0.64 and 0.92 mmol g^–1^ Pd, respectively, according to XPS analysis.

Magnified XPS spectra of both MWCNT-L1-Pd and G-L2-Pd in the N 1s range (Figure 3) show signals with three components. One of them, corresponding to the nitrogen atoms of the conjugate pyrimidine moiety (400.25 eV, L1; 400.15 eV, L2) is placed at the same BE of the corresponding hybrid (i.e., MWCNT-L1 and G-L2, respectively). A second component placed at the lowest BE energy (399.2 eV, MWCNT-L1-Pd; 398.9, G-L2-Pd) corresponds to noncoordinated amino groups (two of the pyrimidine rings in L1, two aliphatic plus one in L2). The third component is placed at the highest BE energy and contains the three Pd-coordinated amino groups of the macrocyclic ring as illustrated in Figure 3. These data show that (i) the macrocyclic ring is the prevalent adsorption site for Pd(II) ions, giving rise to the formation of 1/1 polyamine complexes, and (ii) this coordination mode does not affect the interaction of the ligand pyrimidine moieties with the graphene surface of the carbon supports.

In accordance with the above, the component of the O 1s signal of the catalysts corresponding to C(5)–NO and C(6)=O groups of the pyrimidine moiety of the adsorbed ligands also remains unaltered with respect to the corresponding precursors MWCNT-L1 and G-L2 (Figure S6)

**Figure 4:**
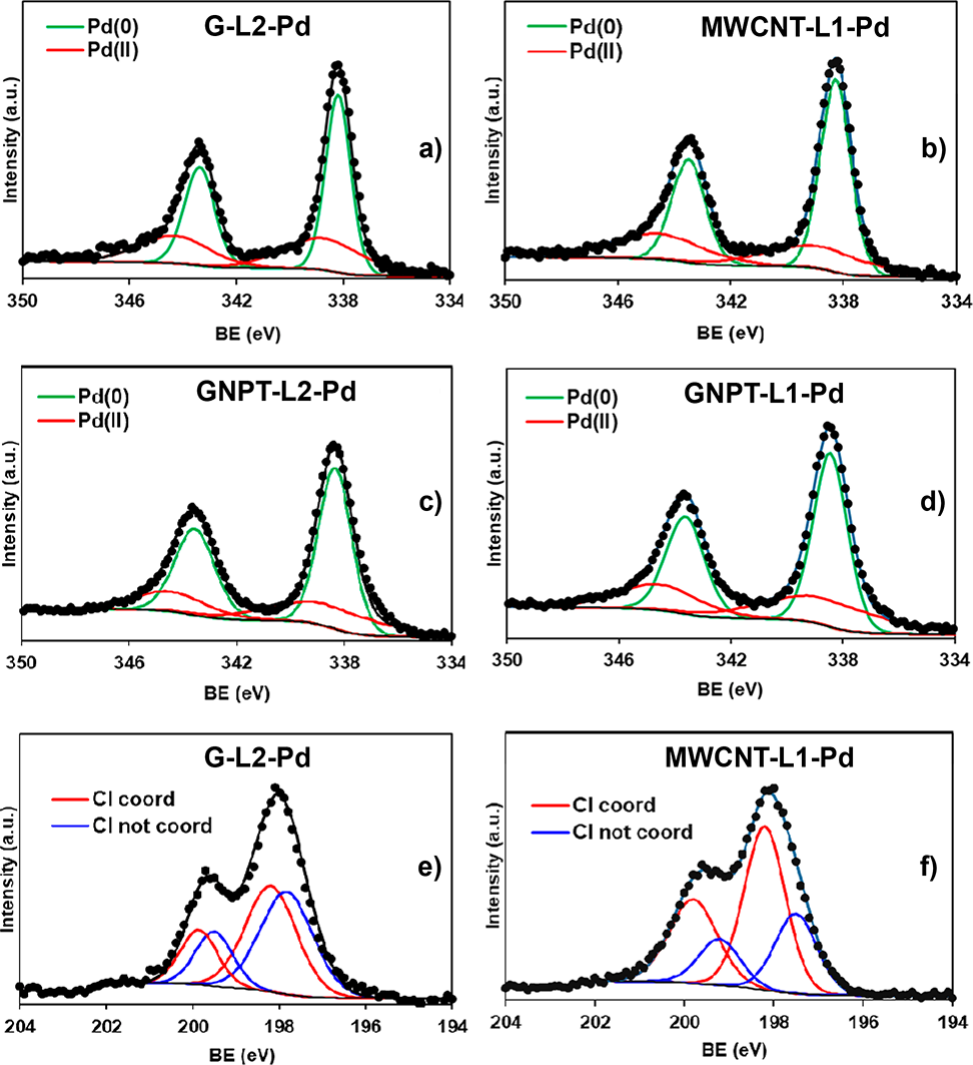
High-resolution XPS spectra of the catalysts in the (a–d) Pd 3d_5/2_ and Pd 3d_3/2_ regions and in the (e, f) Cl 2p_3/2_ and Cl 2p_1/2_ regions.

The coordination pattern of Pd(II) to the polyamine ring of the ligands would require two different moles of Cl^–^ per mole of adsorbed Pd(II), one to complete the square-planar coordination environment of Pd(II) and one, a free chloride anion, to ensure the charge neutrality.^36,37^ XPS spectra of both MWCNT-L1-Pd and G-L2-Pd (Figure 4) show a signal in the Cl range consisting of two peaks which are assigned to the 2p_3/2_ and 2p_1/2_ states of Cl. Each peak consists of two components corresponding to coordinated (the higher-energy one) and noncoordinated (the lower-energy one) Cl^–^ anions.

**Figure 5:**
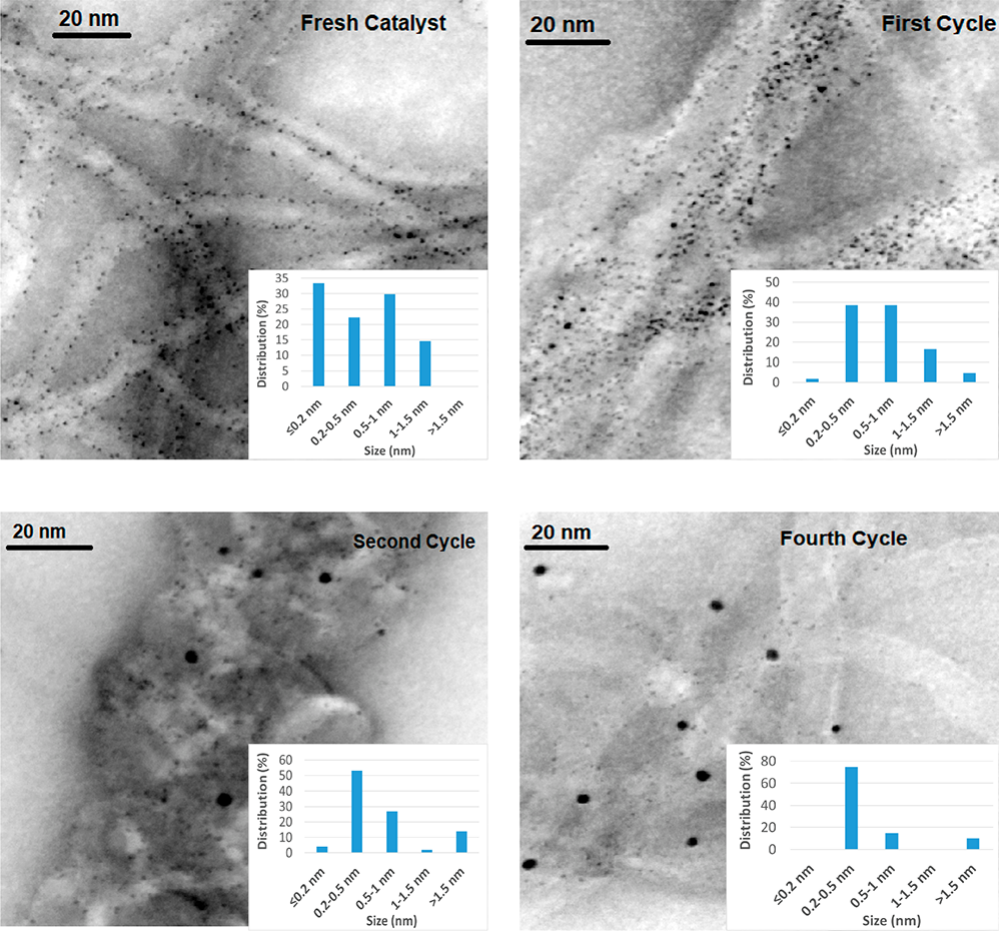
TEM micrographs of fresh and reused MWCNT-L1-Pd catalysts.

Analytical data obtained from XPS analysis indicate that MWCNT-L1-Pd and G-L2-Pd contain an excess of chlorine with respect to the amounts required for the Pd(II) coordinated to L1 and L2. This is not surprising because it is known that PdCl_4_^2–^ can be adsorbed via C–dπ interactions with the arene centers of these sorbents.^62,63^ Nevertheless, the excess Cl is much less than what would be required if the entire excess of Pd would have been adsorbed as PdCl_4_^2–^. An explanation for this observation came from the analysis of TEM micrographs of the two materials (Figure 5 and Figure S7) which showed the presence of a significant amount of Pd(0), in the form of Pd(0) nanoparticles (Nps) deposited on the graphene surfaces, probably generated by photoreduction of part of the adsorbed PdCl_4_^2–^. Further evidence of the presence of Pd(0) in MWCNT-L1-Pd and G-L2-Pd was provided by the XPS spectra in the Pd 3d region showing two signals each containing two components: a minor one corresponding to Pd(II), at ca. 345 eV, and a major one corresponding to Pd(0), at ca. 339 eV.

According to these observations, there are three different palladium species on the surfaces of the above materials: a polyamine-Pd(II) complex (L1-Pd(II) or L2-Pd(II)), PdCl_4_^2–^, and Pd(0) Nps.

### Catalytic Activity of MWCNT-L1-Pd and G-L2-Pd in the Copper-Free Sonogashira C–C Coupling Reaction

The use of MWCNT-L1-Pd in the copper-free Sonogashira reaction of iodobenzene (IB) with phenylacetylene (PA), carried out under conditions similar to those previously adopted for G-L1-Pd (Experimental Section),^37^ provided the maximum conversion to diphenylacetylene (DPA) of 91% in the equilibrium time of 7 h. The maximum conversion obtained in the case of G-L1-Pd was very similar (90%), but the equilibrium time was significantly longer (14 h). According to its structure (see above section), the shorter equilibrium time of MWCNT-L1-Pd could be attributed to the location of Pd active centers at the external surface of MWCNTs, which aids in the easier diffusion of reactants and products compared to the case of G-L1-Pd. As a matter of fact, for similar G-based photocatalysts, the aggregation of functionalized-G sheets was found, probably favored by hydrogen bonding between the F residues of Ar-S-F molecules of adjacent G-Ar-S-F sheets which reinforce vdW dispersion forces.^52^ In the case of G-L1-Pd, stacking remains after the adsorption of Pd, probably because of a sort of “Velcro effect” between adjacent sheets promoted by the hanging functionalities. Such aggregation is expected to hinder the diffusion of species toward/away from the active catalytic centers (Pd ions/atoms), thus determining longer equilibrium times. In this regard, another important factor is the adsorbability of substrates on the carbon supports. In this case, IB is significantly adsorbable on G under the reaction conditions (8% mol), but it is not adsorbed at all on MWCNT.

**Figure 6:**
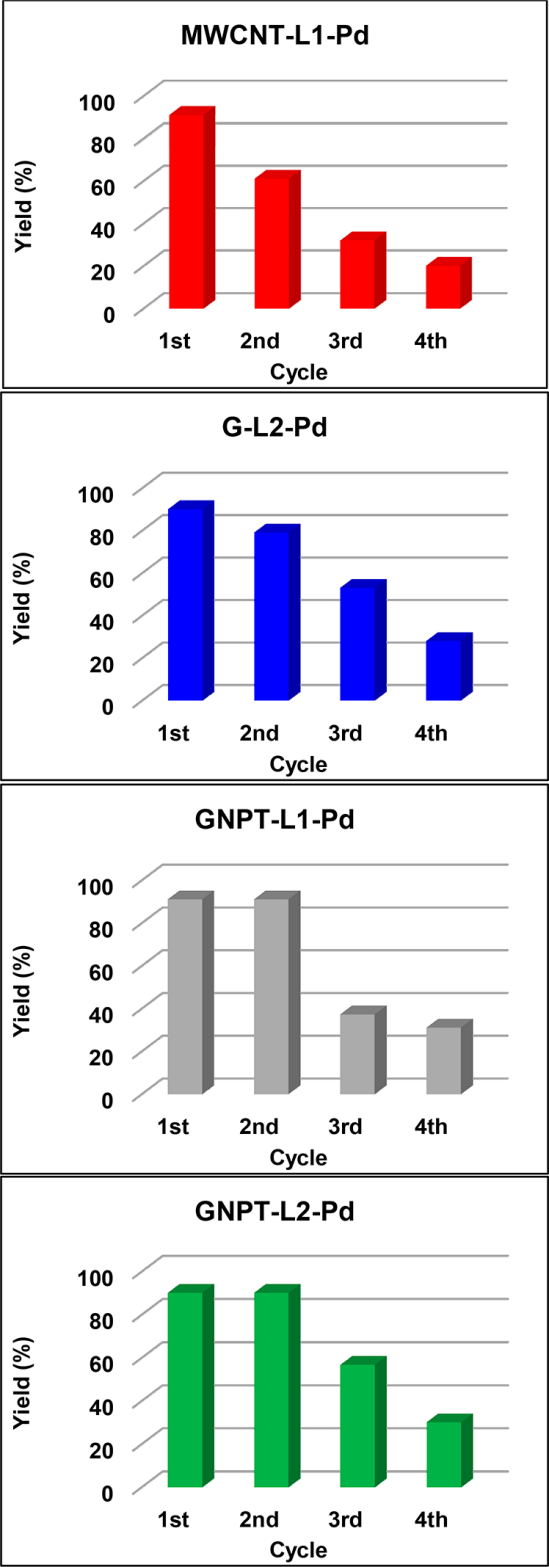
Reaction yields (referring to diphenylacetylene formation) for fresh and reused catalysts.

After the first reaction cycle, MWCNT-L1-Pd was recovered and then reused in three additional cycles. For comparison with the previously studied G-L1-Pd catalyst,^37^ the yield in each cycle was obtained after 7 h. However, catalysts reuse may involve structural changes that affect equilibrium times with respect to the fresh catalyst. The yields with MWCNT-L1-Pd decrease steadily to 62.0% (second cycle), 32.0% (third cycle), and 20% (fourth cycle) (Figure 6), showing greater deactivation than in the case of G-L1-Pd (69, 63, and 50% after 7 h, ref (37)).

**Figure 7:**
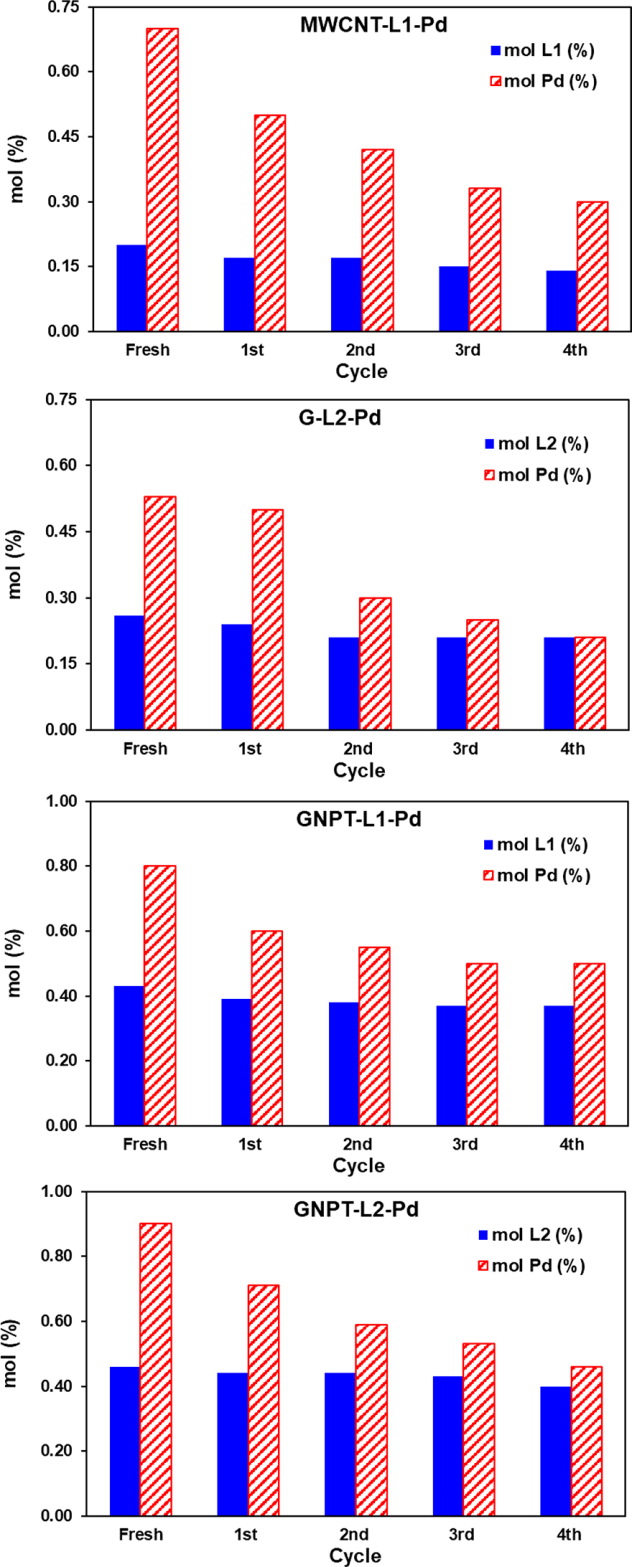
Molar percentages of ligands and Pd in the fresh and reused catalysts (obtained from XPS data).

Information on the origin of such deactivation of MWCNT-L1-Pd after reuse can be gained from the analysis of molar amounts of L1 (N % atom) and Pd (Pd % atom) in the fresh and reused catalysts that can be derived from the % atom composition provided by the corresponding XPS spectra (Figure 7). N decreases slightly but continuously as the catalyst is reused in the four cycles (about a 31% loss, Figure 7), indicating a significant loss of L1-Pd. This is higher than for the G-L1-Pd analogue, for which N remained nearly constant after reuse,^37^ denoting that the interaction of L1 with the flat surface of G is stronger than that with the curved surface of MWCNTs. On the other hand, the % loss of Pd is faster than that of N in the first three cycles of reuse but becomes the same in the fourth cycle (Figure 7), indicating that the loss of Pd during the latter is mostly due to the loss of L1-Pd. The molar Pd/N atom ratio after the fourth cycle is ca. 2.07. This means that 34% of Pd of the fresh catalyst (Pd/N = 3.04) is easily lixiviated, which is the main cause of the strong loss of catalyst activity during reuse.

The polyamine component of the N 1s signal in the XPS spectrum of the catalyst remained unaltered during reuse, indicating that the polyamine ligand contributes to the stabilization of the Pd remaining in the catalyst after each cycle. Thus, the loss of activity in the first three reaction cycles is reasonably borne by the Pd(0) nanoparticles and by the PdCl_4_^2–^ initially deposited on the outer surface of MWCNTs (see section above), with which they interact weakly, and only minor Pd loss is due to the desorption of L1-Pd. Conversely, the much smaller activity loss observed in the fourth cycle is entirely due to a modest L1-Pd desorption.

**Figure 8:**
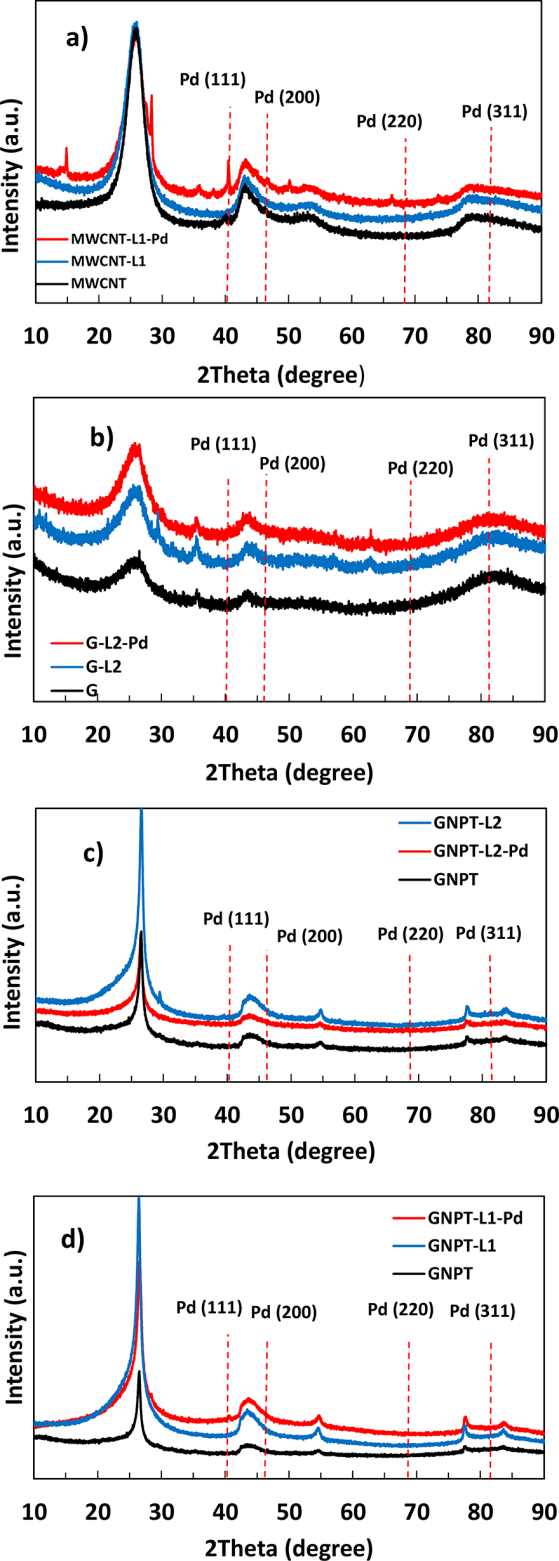
XRD diffraction patterns of (a) MWCNT, MWCNT-L2, and MWCNT-L2-Pd; (b) G, G-L2, and G-L2-Pd; (c) GNPT, GNPT-L2, and GNPT-L2-Pd; and (d) GNPT, GNPT-L1, and GNPT-L1-Pd.

TEM images of fresh and reused catalysts show that a significant number of very small Pd(0) Nps are retained in the internal tubes of the MWCNTs (Figure 5). Most of the particles having dimensions smaller than the diameter of the internal tubes (<5 nm)^36^ are retained during the reaction cycles, while some of the very small ones (≤0.2 nm) are probably lost during the former cycles. Accordingly, plots of the quantities of Pd(0) Nps versus the corresponding sizes in fresh and reused catalysts (Figure 5) clearly illustrate that Nps with a size of 0.2–0.5 nm become the most abundant in the successive reaction cycles. Furthermore, it is also seen that Nps larger than 0.5 nm, placed at the external surface of the MWCNTs, tend to agglomerate as the number of cycles increases. This agglomeration phenomenon is also responsible, together with the loss of Pd, for the decay of the catalyst activity through reuse.^22^ The XRD spectrum of the fresh MWCNT-L1-Pd catalyst (Figure 8) shows the peaks of the bare MWCNT and those at 2θ values of ca. 40° and ca. 46° (weak) (JCPDS no. 46-1043) due to Pd(0) Nps. Pd(0) peaks are observed despite the relatively low amount of Pd(0) in the sample (less than 5%, according to composition and structure data)^64^ probably because most of the Pd(0) Nps are placed on the external surface of the catalyst, where they are easily accessible to radiation. On the contrary, these peaks are unobserved in the corresponding XRD spectra of the reused catalyst (Figure S8).

The progressive reduction of Pd(II) to Pd(0) is observed by examining the Pd signals in the XPS spectra of the reused catalysts (Figure S9). This is commonly observed in Sonogashira C–C coupling reactions catalyzed with Pd complexes and can be responsible for some loss of activity of the reused catalysts. In our case, however, this effect is attenuated probably because of the stabilization of the Pd(0) Nps generated during the reactions provided by the polyamine residue of L1.^22,65−68^

The fresh G-L2-Pd catalyst also shows a high catalytic efficiency (90% DPA yield after 7 h of reaction) which is slightly lower than that obtained when MWCNT-L2-Pd was used in the same reaction (94% in 2 h, ref (36)). This could be explained by assuming that, similar to what was previously observed for G-Tren and G-Tren-Pd materials,^52^ the stacking of G-L2-Pd sheets makes the diffusion of the reagents toward the active sites slower than toward the more exposed active sites of MWCNT-L2-Pd (see above). As a matter of fact, in the XRD spectra of G-L2 and G-L2-Pd, the peaks due to diffraction in the slits between sheets at 2θ = 26.14 show significantly higher intensity than in G^52^ (Figure 8), in agreement with the above-mentioned stacking. It is worth mentioning that these peaks, corresponding to bare G, are the only ones appearing in the XRD spectra of both G-L2 and G-L2-Pd. None of the diffraction peaks expected from Pd(0) face-centered cubic crystals (at 2θ values of 40.05, 46.60, 68.14, and 82.04°; see JCPDS no. 46-1043) are observed in the XRD spectrum of G-L2-Pd in Figure 8. Most likely, the low Pd(0) content in the fresh catalyst (8.2% weight calculated, keeping in mind both the composition and the above structural data) and its inner location in the sample structure prevent the corresponding peaks from appearing clearly defined.^64^

For comparative purposes, the catalytic activity in the Sonogashira reaction of G-L2-Pd after a reaction time of 7 h in three additional cycles was also studied. Similar to the case of the MWCNT-L1-Pd catalyst, the reuse of G-L2-Pd resulted in a continuous loss of catalytic activity: 80% (second cycle), 53% (third cycle), and 30% (fourth cycle). Despite almost half of the initial amount of Pd being lost after the third reaction cycle, these data show a lower loss of activity than in the case of MWCNT-L1-Pd (20% conversion in the fourth cycle) (Figure 6). Two facts can justify this behavior: (i) desorption of the complex during reuse is lower for G-L2-Pd (ca. 18%) than for MWCNT-L1-Pd (ca. 31%) and (ii) the excess Pd (molar ratio Pd/ligand = 2 in this catalyst) is much lower than in the case of MWCNT-L1-Pd (see above). As shown in Figure 7, the Pd/ligand molar ratio decays sharply during the first three reaction cycles and then remains almost constant, equal to 1, after the fourth cycle. This fact indicates that once the excess Pd is lost, the remaining Pd is retained thanks to the stabilization provided by the macrocyclic function of L2. In this regard, the nitrogen percentages of the reused catalysts remain practically unchanged after each of the reaction cycles, confirming the stabilizing role of the macrocyclic function on Pd retained during reuse (Table S7).

Figure S7 shows high aggregation of Pd(0) Nps on the surface of fresh G-L2-Pd, indicating that the large molar excess of Pd, relative to L2, is weakly retained, thus favoring the observed aggregation. As shown in the Figure S7, a smooth loss of Pd(0) affecting the larger particles takes place after the first reaction cycle, which results in a decrease in the average size of the remaining Pd(0) nanoparticles. Accordingly, similar to the case of the MWCNT-L1-Pd catalyst, TEM images of the reused G-L2-Pd catalyst show little, albeit progressive, aggregation of Pd(0) Nps in the successive reaction cycles, up to a mean size that approximately fits the slit between sheets (0.49 Å)^52^ (Figure S7).

Moreover, a progressive reduction of Pd(II) to Pd(0) in the catalysts (mainly coordinated to the polyamine function of L2) also occurs through reuse (Figure S9). These two occurrences also contribute to the loss of activity described above. As already mentioned,^22,65−68^ Pd(II) reduction is commonly observed in the copper-free Sonogashira reaction with catalysts based on Pd(II) complexes and also with catalysts similar to those used here.^36,37,53^ Similar to what was observed for the fresh G-L2-Pd, no Pd(0) peaks appeared in the XRD spectrum of the reused catalysts, which prevents the acquisition of additional information on the structure of Pd Nps (Figure S8) (see above).

The much smaller desorption of the complex from G-L2-Pd than from the previously studied MWCNT-L2-Pd (approximately 50% in four reaction cycles)^36^ and from MWCNT-L1-Pd (see above) can be attributed to a much stronger interaction of the pyrimidine group of L2 with the flat surface of G than with the curved surface of MWCNTs. Furthermore, it could be thought that the packed-sheet structure of G-L2-Pd, while delaying the equilibrium time, may favorably hinder the desorption of the complex.

In summary, the adopted supramolecular approach to the surface functionalization of graphene supports allows a uniform spreading of the catalytically active Pd centers, which correlates with the efficiency of the studied catalysts. Indeed, MWCNT-L1-Pd and G-L2-Pd, which contains PdCl_4_^2–^, Pd(0) Nps, and Pd(II) ions coordinated to the corresponding polyamine functions, render high yields in the above-mentioned Sonogashira copper-free C–C coupling reaction in one reaction cycle. However, the reuse of the catalysts in additional reactions determines a rapid deactivation as Pd(0) and PdCl_4_^2–^, also endowed with catalytic activity, undergo almost total lixiviation during the first cycles because of their weak interaction with the graphene surfaces. On the contrary, the strong interaction of L1 and L2 with the Cπ centers of the carbon supports, together with their ability to form highly stable Pd(II) complexes, determines that most of these polyamine-Pd centers remain unaltered and maintain catalytic activity through reuse. The data obtained in this work, together with previous results,^36,37^ also show a general tendency of the pyrimidine group (Ar) of Ar-S-F ligands to have stronger interactions with G than with MWCNTs, providing greater stability (i.e., better catalytic performance) to functionalized G materials. Thus, flat graphene-based supports are more suitable than MWCNTs for the design of catalysts, of the above type, for Sonogashira C–C coupling reactions.

### Catalytic Activity of GNPT-L1-Pd and GNPT-L2-Pd in the Copper-Free Sonogashira C–C Coupling Reaction

Within this scenario, we have extended the study to the analysis of the catalytic performance of two hybrid materials based on graphene nanoplatelets (GNPT), GNPT-L1-Pd and GNPT-L2-Pd, with the aim of acquiring information on the possible influence of the dimensions of the graphene sheets on the behavior of this type of catalyst. Graphene nanoplatelets are a kind of graphene consisting of stacks of few graphene sheets (<10) with a size smaller than graphene.

As observed for L1 and L2 as well as for another ligand^52^ containing a TREN (tris(2-aminoethyl)amine) unit linked to the same pyrimidine residue, adsorption on G promotes the stacking of functionalized G sheets. Moreover, studies on the acid–base properties of these materials suggest that the stacked-sheet structures observed in the solid phase are retained when they are suspended in water.^36,37^ Even the further adsorption of PdCl_4_^2–^ on the ligand-G hybrids does not affect the stacked structure. Thus, during the catalytic reaction, most of the active Pd centers of the G–Ar–S-F-Pd catalysts remain in the very narrow pores between the stacked sheets.

Both GNPT-L1-Pd and GNPT-L2-Pd were prepared using, as a carbon support, commercial GNPTs with an average diameter (<1.5 μm) 5 times lower than that of G.^54^ The preparation was carried out by following a two-step procedure similar to that described in the previous section for MWCNT-L1-Pd and G-L2-Pd. Notably, the maximum adsorption capacities of L1 and L2 on the used GNPTs are significantly higher than those on G, especially in the case of L2 (L2, 0.82 mmol g^–1^; L1, 0.55 mmol g^–1^). This is noteworthy because the available surfaces of similar masses of both graphene sorbents, having similar number of sheets/unit, should be similar: the observed differences suggest that the graphene surface of G is less efficiently covered by ligand molecules than that of GNPTs.

This result can be tentatively explained by assuming that the stacking of adjacent functionalized graphene sheets occurs before the adsorption equilibrium is reached, thus limiting the amount of ligand adsorbed. In that case, it would be reasonable for smaller GNPT sheets to limit adsorption less than that for larger G sheets. Anyway, the higher adsorptivity of ligands on GNPTs offers a considerable advantage because it leads to obtaining better catalysts with a greater density of active sites.

The GNPTs sample that was used contains C (92.2% by weight) and O (7.8% by weight) as determined from XPS spectra (Figure S1). The C 1s high-resolution XPS spectrum shows the components of the aromatic sp^2^ C atoms (at ca. 284.6 eV) and of those attached to oxygen (epoxy and hydroxyl functions at ca. 285.6 eV), carbonyl (at 287.3 eV), and carboxyl functions (at 289.0 eV). The O 1s high-resolution spectrum consists of a wide signal with a maximum at ca. 533 eV, which contains the oxygen atoms from different functions: epoxy and hydroxyl groups at 533.5 eV, carbonyl functions at 532.2 eV, and carboxylic functions at 531.9 eV (Figure S1).

Adsorption on the GNPT-L1 and GNPT-L2 hybrids of PdCl_4_^2–^ from aqueous solutions (Experimental Section) allowed us to obtain GNPT-L1-Pd and GNPT-L2-Pd materials with Pd/L1 and Pd/L2 molar ratios equal to 1.86 and 1.95, respectively.

**Figure 9:**
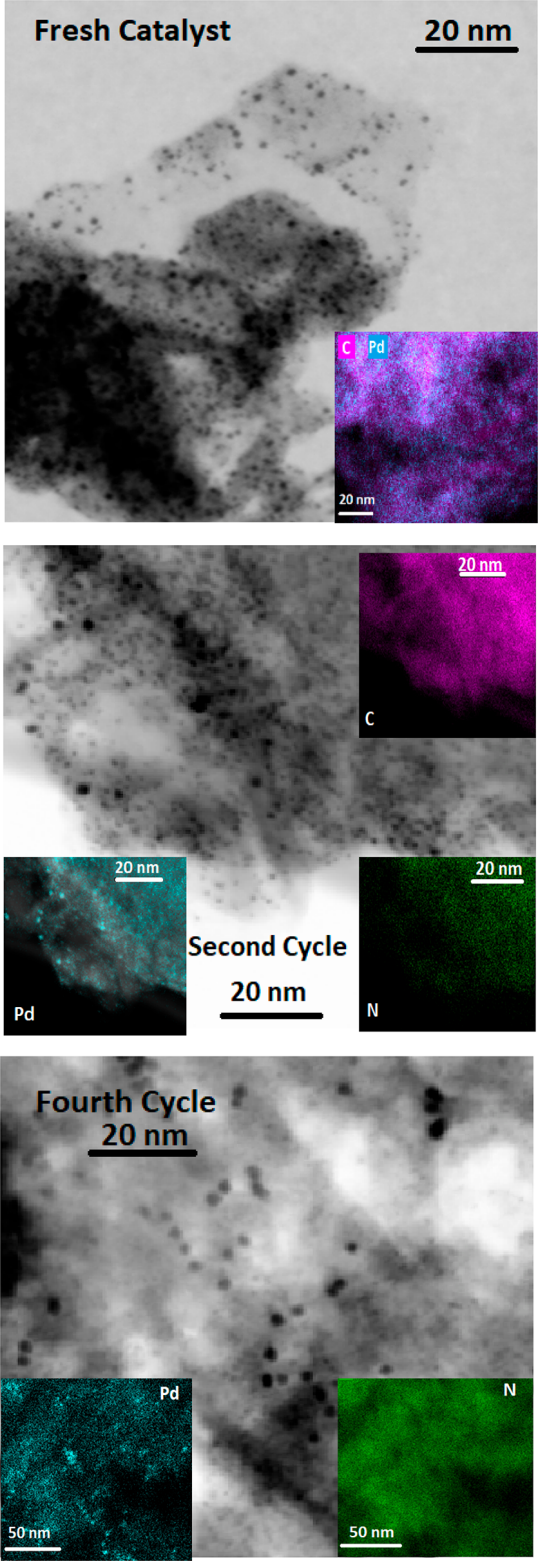
TEM micrographs of GNPT-L2-Pd: (top) fresh catalyst; (middle) after the second cycle; and (bottom) after the fourth cycle. Insets: element distribution maps.

Similarly to the cases of the MWCNT and G adsorbents, the N 1s and O 1s components of the high-resolution XPS spectra of the free ligands are shifted to higher BE values in GNPT-L1 and GNPT-L2 (Figures 3 and S10), in agreement with the interaction of the pyrimidine moiety of the ligands with the Cπ centers of the graphene surface. Successive adsorption of PdCl_4_^2–^ on the GNPT-L1 and GNPT-L2 hybrids causes a shift toward higher BE of the aromatic components of the N 1s signals in the corresponding XPS spectra, indicating that the ligand pyridine group is involved in metal coordination; that is, the macrocyclic polyamine functions of the adsorbed L1 and L2 ligands are the prevalent coordination sites for Pd(II), determining the formation of 1:1 metal/ligand complexes (see above). The chlorine contents of GNPT-L1-Pd and GNPT-L2-Pd obtained from XPS spectra (Table S7) show that, in both materials, the excess adsorbed Pd with respect to ligands (0.37 and 0.44 mmol g^–1^, respectively) is mostly in the form of Pd(0) whereas only a very small amount (ca. 10 % atoms) is in the form of PdCl_4_^2–^. This result is consistent with the TEM images of both catalysts (Figures 9 and S11) showing significant numbers of Pd(0) nanoparticles uniformly distributed on their surfaces. In accordance with this, the Pd 3d_5/2_ and Pd 3d_3/2_ components in the XPS spectra (Figure 4) consist of asymmetric signals resulting from the Pd(II) and Pd(0) species existing on the surface of the graphene sheets of both catalysts.

Regarding the textural properties, it is worthy of mention that, similar to what has been observed for analogous G-based materials,^52^ GNPTs undergo an important decrease in the pore volume in the range of mesopores (from 0.933 cm^3^ g^–1^ in GNPTs to 0.563 cm^3^ g^–1^ in GNPTs-L1) and in the BET surface area (from 668.4 to 156.4 m^2^ g^–1^) as a consequence of the functionalization with L1. As already mentioned, this is due to the ability of GNPT-L1 sheets to form stacks, probably favored by hydrogen bond interactions between the polyamine functions. Interestingly, the stacking was preserved after the adsorption of PdCl_4_^2–^, which means that most of the potential catalytic active sites are placed in the very narrow pores formed between the stacked sheets. Stacking gives rise to a significant increase in the intensity of the peak at ca. 27 (2θ) in the XRD spectra of both GNPT-L1 and GNPT-L1-Pd with respect to GNPT (Figure 8). Similar behavior is observed when comparing the XRD spectra of GNPT and GNPT-L2, indicating a greater aggregation of sheets in the latter than in nonfunctionalized GNPT. Nevertheless, the intensities of the peaks in the XRD spectrum of GNPT-L2-Pd are lower than in the GNPT-L2 spectrum (Figure 8), indicating that some disaggregation occurs after the adsorption of PdCl_4_^2–^. According to the above hypothesis, this should indicate that the cooperative binding of Pd by ligand molecules of adjacent sheets is less significant in the case of L2.

TEM images of GNPTs-L1-Pd (Figure S11) and GNPTs-L2-Pd (Figure 9) show a significant aggregation of sheets in agreement with the above conclusions. The distribution of elements on the surfaces of these catalysts (Figures 9 and S11) shows that both ligands (L1 and L2) are much more uniformly distributed on the surface of the carbon support than Pd, which exhibits different irregular distributions depending on the different forms in which it is adsorbed.

The catalytic activities in the studied Sonogashira reaction of both GNPT-L1-Pd and GNPT-L2-Pd fresh catalysts for a 7 h equilibrium time are similar (91.0% for GNPT-L1-Pd and 90.2% for GNPT-L2-Pd based on the DPA formed). According to these values, the GNPT-based catalysts work very efficiently and equal the catalytic performance of G-L2-Pd and MWCNT-L1-Pd, which is consistent with the very similar nature of their catalytically active centers that comprise polyamine-Pd(II) complexes, minor amounts of PdCl_4_^2–^, and Pd(0) nanoparticles.

After the first reaction cycle, the catalysts (GNPT-L1-Pd and GNPT-L2-Pd) were recovered and then reused in three additional reaction cycles. For comparison purposes, the results obtained from these experiments after 7 h of reaction are shown in Figure 6 together with the analogous data for MWCNT-L1-Pd and G-L2-Pd. Although the equilibrium times for this type of catalyst become longer as the number of reaction cycles increases, the kinetic data at 7 h reaction times are useful for comparing the performances of the catalysts.

It is apparent in Figure 6 that GNPT-based catalysts also show a reduction of activity after repeated use, although the loss is significantly less than for both G-based and MWCNT-based catalysts. In the first reaction cycle, all catalysts exhibit similar efficiencies, but the catalytic activities of G-based and MWCNT-based ones steadily decay after the first reaction cycle whereas GNPT-based counterparts preserve their original activities during the second cycle and suffer a smaller loss in successive ones (Figure 6).

These activity data can be analyzed in light of analytical and structural properties of the catalysts. Quantitative analysis of N percentages from survey XPS spectra of each catalyst provides the data shown in Figure 7. A nitrogen loss of about 15% occurs in the case of the two GNPT-based catalysts during the first two reaction cycles, but then the content remains constant. These losses are lower, or much lower, than in the cases of G-L2-Pd (20% N loss during the first three reaction cycles) and MWCNT-L1-Pd (31% constant N loss during the four reaction cycles). Thus, as far as the ligand-adsorbent interaction is concerned, the GNPT-based catalysts are more stable than those based on MWCNT and G.

Figure 7 also shows Pd percentages in the fresh and reused catalysts obtained from the corresponding survey XPS spectra. As in the case of G-L2-Pd and MWCNT-L1-Pd, GNPT-based catalysts also suffer significant Pd loss during reuse. This occurs through the first two reaction cycles, after which the Pd/ligand molar ratio remains almost constant at values greater than 1 (notably 1.35 for GNPT-L1-Pd and 1.15 for GNPT-L2-Pd after the fourth reaction cycle). This is in contrast to what was observed for G-L2-Pd, which gave a Pd/ligand molar ratio equal to 1 after the fourth cycle. As discussed before, excess Pd over the 1:1 Pd/ligand molar ratio is adsorbed in the forms of PdCl_4_^2–^ and Pd(0). These species, which interact weakly with the graphene surface, undergo easier lixiviation than the Pd(II) ions coordinated to the macrocyclic polyamine units, and the distribution of Pd becomes more and more similar to that of the ligand in successive catalytic cycles (Figure 9). Therefore, the ability of GNPT-based catalysts to retain more Pd (especially in the case of GNPT-L1-Pd, see above) during reuse could be attributed to both a trapping effect given by their close packing of stacked sheets and to their higher surface density of polyamine molecules.

The composition and the stacked-sheet structure of both GNPT-based materials remain nearly constant after the second cycle. Moreover, the reduction of Pd(II) in the case of reuse is insignificant compared to that of the aforementioned catalysts based on G and MWCNTs (Figure S9). Despite this, Figure 6 shows the loss of activity in both GNPT-based catalysts during the last two cycles, which can mainly be attributed to some aggregation of Pd(0) nanoparticles. Although TEM analysis of fresh and reused GNPT-L1-Pd and GNPT-L2-Pd provided poorly defined images of Pd(0) Nps, we performed a tentative analysis of the corresponding distribution sizes (Experimental Section). Although this analysis was limited to a small number of Nps, the results reveal that Pd(0) Nps in both fresh catalysts have significantly lower mean sizes (Figures S12 and S13) than in the case of the fresh G-L2-Pd catalyst. This could be due to the higher surface density of L1 and L2 in both GNPT-based catalysts, relative to G-L2-Pd, which limits the aggregation of Pd(0) Nps in the former more than in G-L2-Pd. After the first cycle, the reuse of both GNPT catalysts results in a modest aggregation of Pd(0) Nps, indicating higher stability than in the cases of MWCNT-L1-Pd and G2-L2-Pd.

Similarly to the case of fresh and reused G-L2-Pd catalysts, the lack of any XRD peaks characteristic of Pd(0) crystals in the corresponding spectra (Figure S8) of fresh and reused GNPT-based catalysts prevents further analysis of the Pd(0) Nps.

## Conclusions

This work provides some key inputs to improve the design of heterogeneous catalysts for Sonogashira copper-free reactions based on graphene supports bearing Pd as active catalytic centers.

First, we remark that the supramolecular (noncovalent) and environmentally friendly approach used for the functionalization of these supports is confirmed to be very effective for the production of robust and efficient catalysts despite the weak forces involved in critical stages of their assembly.

The new heterogeneous catalysts, based on G, GNPT, and MWCNT supports, were prepared in such a way as to obtain an overall load of Pd greater than that corresponding to the coordination of Pd(II) to the macrocyclic functions of L1 and L2, with the excess metal being adsorbed as Pd(0) Nps and PdCl_4_^2–^. This allowed for the simultaneous analysis of the stability and catalytic efficiency of three different types of metal centers on three different types of carbon supports in the presence of two ligands with different anchoring capabilities.

Using the copper-free Sonogashira reaction between iodobenzene and phenylacetylene to produce diphenyalacetylene as a test, all catalysts obtained by anchoring on the three supports of Pd(II) complexes of L1 and L2 were shown to have similar good performances in one reaction cycle, whereas catalysts based on G and GNPT provide greater catalytic efficiency in reuse.

The conservation of catalytic efficiency during reuse of the catalysts depends on the chemical nature and the stability of the active centers of Pd because the deactivation occurs mainly through the loss of Pd. Compared to the Pd(0) Nps and PdCl_4_^2–^ active centers deposited directly on graphene-type supports, which lixiviate easily, graphene-based catalysts functionalized with Ar-S-F-type ligands provide robust catalytic Pd(II) centers firmly coordinated to the F ligand functions. Nevertheless, progressive Pd(II) reduction from these Pd(II) complex active centers during reuse gives rise to some loss of activity, although such a phenomenon is greatly attenuated via Pd complexation of the deposited Pd(0) Nps by the polyamine F functions.

Thus, the higher deactivation on reuse of the catalysts studied in this work, compared to others previously studied in which all active sites (Pd(II) ions) were complexed by the polyamine ligand functions,^36,37^ is due to the easy lixiviation of PdCl_4_^2–^ and Pd(0) centers from the formers.

Another important issue concerning the stability of the active Ar-S-F-Pd centers on the graphene-based surface is related to the anchoring strength of the Ar-S-F ligands. In this regard, the flat surface of G and GNPT supports is more suitable for an efficient anchoring than the curved surface of MWCNTs. An important finding regarding the stability (i.e., the catalytic efficiency of the graphene-based catalysts bearing polyamine functions L1 and L2; the same can be said for the previously studied ligand Tren supported on G)^52^ is the tendency toward spontaneous stacking of Pd derivatives of both functionalized G and GNPT sheets. The resulting close-packed structures provide greater robustness to the catalysts, which resulted in a smoother activity loss when reused in the studied catalytic reactions. In this regard, it is more advantageous to use GNPT rather than G as a carbon support. The smaller size of GNPT sheets compared to G sheets favors the presence of more ligand molecules per unit area on GNPT than on G. This results in a smaller quantity of the carbon material necessary for the preparation of the catalysts and, in the case of L2, leads to a greater robustness and catalytic efficiency of GNPT-L2-Pd compared to G-L2-Pd.

The set of results of this work, together with others obtained previously,^36,37^ encourages the study of the behavior of similar catalysts in Sonogashira reactions with other types of substrates, of both flat and nonflat structures. This would allow a deeper understanding of the influence of the structures of both the tertiary carbon-ligand-Pd hybrid material and the ligand-Pd complex representing the catalytically active centers. Moreover, the results also encourage the extension of this study to the behavior of catalysts of the Ar-S-F-Pd type (Ar = graphene) in the Sonogashira reaction with a broad range of other halobenzene and phenylacethylene derivatives.

## Supporting Information Available

Crystal data and refinement parameters, tables of bond distances and angles, crystal structure of **2**, XPS spectra and TEM micrographs of the catalysts, NMR spectra of reaction products, textural characteristics of GNPT and GNPT-L1, nitrogen adsorption and desorption isotherms for GNPT and GNPT-L1, and X-ray powder diffraction spectra (PDF)
