# Supplementary material for: Noncovalent Assembly and Catalytic Activity of Hybrid Materials Based on Pd Complexes Adsorbed on Multiwalled Carbon Nanotubes, Graphene, and Graphene Nanoplatelets
Source: Inorg Chem. 2022 Aug 4;61(32):12610–24. doi: 10.1021/acs.inorgchem.2c01559 (PMC9387097; doi:10.1021/acs.inorgchem.2c01559)
Supplement: Supplementary file 1 — ic2c01559_si_001.pdf [file ic2c01559_si_001.pdf]

Supporting Information

Noncovalent Assembly and Catalytic Activity of Hybrid Materials Based on Pd Complexes Adsorbed on Multiwalled Carbon Nanotubes, Graphene, and Graphene Nanoplatelets

Alba M. Valbuena-Rus,[†] Matteo Savastano, [‡,§] Paloma Arranz-Mascarós,[*,†] Carla Bazzicalupi,[‡] María P. Clares,[∥] María L. Godino-Salido,[†] María D. Gutiérrez-Valero,[†] Mario Inclán,[∥] Antonio Bianchi,[*,‡] Enrique García-España[*,∥] and Rafael López-Garzón.[†]

[†] *Department of Inorganic and Organic Chemistry, University of Jaén, 23071 Jaen, Spain. E-mail: parranz@ujaen.es.*

[‡] *Department of Chemistry "Ugo Schiff", University of Florence, Via della Lastruccia, 3-13, 50019 Sesto Fiorentino, Italy. E-mail: antonio.bianchi@unifi.it.*

[§] *National Interuniversity Consortium of Materials Science and Technology (INSTM), Via G. Giusti 9, 50121 Florence, Italy.*

[∥] *ICMol, Department of Inorganic Chemistry, University of Valencia, C/Catedrático José Beltrán 2, 46980 Paterna, Spain. E-mail: enrique.garcia-es@uv.es.*

Content

Table S1. A brief summary illustrating some relevant reactions catalyzed by both metal ion complexes and nanostructured metal compounds on graphene-like surfaces. S2
Table S2. Textural characteristics of GNPT and GNPT-L1. .... S3
Table S3. ^{1}H and ^{13}C NMR chemical shifts for diphenylacetylene (DPA) and Et_3N in $CDCl_3$. S4
Table S4. Crystal data and refinement parameters for $[Pd(HL)Br_2]Br$ and $[HLPdBr_2]Cl_{0.74}Br_{0.26}\cdot H_2O$. S5
Table S5. Bond distances in the coordination environment of $[Pd(HL)Br_2]Br$ and $[Pd(HL)Br_2]Cl_{0.74}Br_{0.26}\cdot H_2O$. S6
Table S6. H-bond distances in $[Pd(HL)Br_2]Br$ and $[Pd(HL)Br_2]Cl_{0.74}Br_{0.26}\cdot H_2O$. S6
Table S7. Chemical composition determined from XPS survey spectra for fresh MWCNT-L1-Pd, G-L2-Pd, GNPT-L1-Pd and GNPT-L2-Pd catalysts and after their use in four catalytic cycles. S7
Figure S1. High resolution XPS spectra of MWCNT and GNPT in the O1s and C1s regions, and survey XPS of MWCNT and GNPT with insets showing the chemical composition of the samples S8
Figure S2. Experimental and calculated XRDP spectra for $[Pd(HL)Br_2]Cl_{0.74}Br_{0.26}\cdot H_2O$. S9
Figure S3. Nitrogen adsorption and desorption isotherms, at 77 K, of GNPT and GNPT-L1. .... S10
Figure S4. ^{1}H NMR (a) and ^{13}C NMR (b) spectra, in $CDCl_3$, of the crude product obtained from the reaction between IB and PA catalyzed by GNPT-L2-Pd. S11
Figure S5. Centrosymmetric dimer of $[Pd(HL)Br_2]^+$ cations in $[Pd(HL)Br_2]Cl_{0.74}Br_{0.26}\cdot H_2O$, H-bonded to the bromide counterions. S12
Figure S6. High-resolution XPS spectra in the O1s region of: a) L1, MWCNT-L1 and MWCNT-L1-Pd; b) L2, G-L2 and G-L2-Pd. S13
Figure S7. TEM micrographs of fresh and reused G-L2-Pd catalyst. S14
Figure S8. XRD diffraction patterns of reused MWCNT-L2-Pd, G-L2-Pd, GNPT-L2-Pd and GNPT-L1-Pd. S15
Figure S9. XPS spectra of fresh and reused (four cycles) catalysts in the Pd 3d region. S16
Figure S10. High resolution XPS spectra of L1, GNPT-L1, L2 and GNPT-L2 in the N1s and in the O1s regions. S17
Figure S11. TEM micrographs of GNPT-L1-Pd: fresh catalyst and after 1/4 cycles with elements distribution maps. S18
Figure S12. TEM micrographs of fresh and reused GNPT-L1-Pd catalyst. S19
Figure S13. TEM micrographs of fresh and reused GNPT-L2-Pd catalyst. S20

Table S1. A brief summary illustrating some relevant reactions catalyzed by both metal ion complexes and nanostructured metal compounds on graphene-like surfaces.

**Nanocatalysts**	**Reactions (experimental conditions)**	**References**
GO/Fe_3O_4/Pd NPs	Reduction of organic dyes: 4-nitrophenol, methyl orange, methylene blue, congo red<br>($NaBH_4$, aqueous medium, room temperature)	10
Cu NPs/rGO	Photocatalytic hydrogen evolution<br>(Lactic acid as sacrificial agent; 300 W Xe lamp, $\lambda \geq$ 420 nm)	14
PyCoPc complex/GO	Electrocatalytic hydrogen evolution<br>(Basic medium, KOH 1M)	15
Cu NPs/rGO	Photocatalytic CO_2 reduction<br>(Halogen lamp, 300 W)	16
CoPc complex /Graphene	Electrocatalytic CO_2 reduction to CO<br>(0.1M $KHCO_3$ electrolyte)	17
Ni NPs/rGO	Cr(IV) reduction<br>($K_2Cr_2O_7$, H_2O/Formic acid, pH 2)	18
Ag NPs/Graphene<br>Ag NPs/MWCNT	Photocatalytic degradation of toxic dyes: rose bengal, methylene blue, and phenol red<br>(300 W lamp, λ = 365 nm)	19
GO/ZnO	Formation of 5-substituted-1*H*-tetrazoles (reaction of nitrile derivatives and NaN_3; 120ºC, DMF, 30h)	11
Mesh-GO/Pd NPs	Stille coupling reaction<br>(90ºC, K_2CO_3, H_2O:EtOH 1:1;<br>Iodobenzene and tributylphenylstannane, 2h<br>Bromobenzene and tributylphenylstannane, 3h)	12
Mesh-GO/Pd NPs	Suzuki-Miyaura coupling reaction<br>(90ºC, K_2CO_3, H_2O:EtOH 1:1;<br>Iodobenzene and phenylboronic acid, 90 min<br>Bromobenzene and phenylboronic acid, 150 min<br>Chlorobenzene and phenylboronic acid, 7h)	12
Pd NPs/Fe_3O_4/GON	Sonogashira coupling reaction<br>(80ºC, NaOAc, DMAc;<br>Iodobenzene and phenylacetylene, 4h)	13, 20

Table S2. Textural characteristics of GNPT and GNPT-L1

Sample	BET surface area (m^2/g)	BJH pore volume (cm^3/g)
**GNPT**	668.4	0.933
**GNPT-L1**	156.4	0.563

Table S3. ^{1}H and ^{13}C NMR chemical shifts (ppm) for diphenylacetylene (DPA) and Et_3N in $CDCl_3$

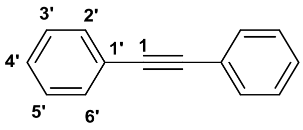

DPA

**Position**	**^{1}H δ/ppm (J/Hz)** $CDCl_3$	**^{13}C δ/ppm** $CDCl_3$
1	-	89.5
1’	-	123.4
2’	7.53-7.50 (*m*)	131.8
3’	7.34-7.31 (*m*)	128.3
4’	7.34-7.31 (*m*)	128.2
5’	7.34-7.31 (*m*)	128.3
6’	7.53-7.50 (*m*)	131.8

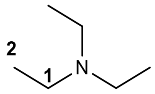

Et_3N

**Position**	**^{1}H δ/ppm (J/Hz)** $CDCl_3$	**^{13}C δ/ppm** $CDCl_3$
1	3.15 (q, *J* = 7.3 Hz)	46.2
2	1.44 (t, *J* = 7.3 Hz)	8.7

Table S4. Crystal data and refinement parameters for $[Pd(HL)Br_2]Br$ (**1**) and $[HLPdBr_2]Cl_{0.74}Br_{0.26}\cdot H_2O$ (**2**).

	(**1**)	(**2**)
Empirical formula	$C_{11}H_{19}Br_3N_4Pd$	$C_{11}H_{21}Br_{2.26}Cl_{0.74}N_4OPd$
Formula weight	553.43	538.55
Temperature (K)	100(2)	293(2)
space group	*P -1*	*P 2₁/n*
a (Å)	7.181(2)	13.6147(6)
b (Å)	9.620(3)	7.5592(4)
c (Å)	11.980(4)	16.1485(8)
α (°)	80.93(1)	90
β (°)	84.62(1)	101.599(5)
γ (°)	71.84(1)	90
Volume (Å^3)	775.6(5)	1628.0(1)
Z	2	4
Independent reflections / R(int)	2741/0.0467	2861/ 0.0491
μ (mm^{-1})/λ(Å)	8.916/0.71073	6.809/0.71073
R indices [I>2σ(I)]*	R1 = 0.0770<br>wR2 = 0.2318	R1 = 0.0350<br>wR2 = 0.0894
R indices (all data)*	R1 = 0.0820<br>wR2 = 0.2364	R1 = 0.0401<br>wR2 = 0.0926
CCDC no.	2161485	2161486

* R1 = $\Sigma \| |Fo| - |Fc| \| / \Sigma |Fo|$; wR2 = = $[\Sigma w(Fo^2 - Fc^2)^2 / \Sigma wFo^4]^{1/2}$

Table S5. Bond distances (Å) in the coordination environment of $[Pd(HL)Br_2]Br$ (**1**) and $[Pd(HL)Br_2]Cl_{0.74}Br_{0.26}\cdot H_2O$ (**2**).

	(**1**)	(**2**)
Pd – Br1	2.415(2)	2.4219(7)
Pd – Br2	2.409(2)	2.4340(8)
Pd – N3	2.06(1)	2.060(5)
Pd – N4	2.05(1)	2.061(4)

Table S6. H-bond distances (Å) in the metal complex dimers of $[Pd(HL)Br_2]Br$ (**1**) and $[Pd(HL)Br_2]Cl_{0.74}Br_{0.26}\cdot H_2O$ (**2**).

(**1**)	(**2**)
N3···Br3 3.22(1)	N3···Cl1 3.215(9)
N4···Br3 3.22(1)	N4···Cl1 3.194(8)
	N3···Br3 3.23(1)
	N4···Br3 3.20(1)

Table S7. Chemical composition (atomic concentration %) determined from XPS survey spectra for the fresh MWCNT-L1-Pd, G-L2-Pd, GNPT-L1-Pd and GNPT-L2-Pd catalysts and after their use in four catalytic cycles (C1-C4).

MWCNT-L1-Pd	C1s	N1s	O1s	Cl2p	Pd3d	I3d
Fresh	88.34	3.23	6.58	1.15	0.70	_
C1	91.94	2.67	4.34	_	0.57	0.49
C2	91.83	1.87	5.73	_	0.33	0.25
C3	92.81	2.33	4.33	_	0.33	0.20
C4	93.85	2.22	3.32	0.07	0.30	0.23
**G-L2-Pd**	**C1s**	**N1s**	**O1s**	**Cl2p**	**Pd3d**	**I3d**
Fresh	87.25	2.63	8.94	0.66	0.53	_
C1	89.98	2.26	6.89	_	0.39	0.48
C2	91.68	2.06	5.47	_	0.29	0.50
C3	92.41	1.83	5.19	_	0.25	0.32
C4	92.29	2.06	5.29	_	0.21	0.15
**GNPT-L1-Pd**	**C1s**	**N1s**	**O1s**	**Cl2p**	**Pd3d**	**I3d**
Fresh	81.59	6.90	9.61	1.11	0.79	_
C1	84.18	6.32	8.46	0.10	0.61	0.33
C2	84.03	6.04	8.92	0.15	0.55	0.32
C3	85.42	5.69	8.05	0.07	0.48	0.29
C4	84.69	5.89	8.54	0.08	0.51	0.29
**GNPT-L2-Pd**	**C1s**	**N1s**	**O1s**	**Cl2p**	**Pd3d**	**I3d**
Fresh	85.47	4.63	7.88	1.14	0.88	_
C1	86.33	4.43	7.90	0.16	0.71	0.48
C2	86.78	4.37	7.83	0.14	0.59	0.30
C3	86.97	4.30	7.82	0.14	0.53	0.24
C4	87.14	3.89	8.15	0.09	0.46	0.27

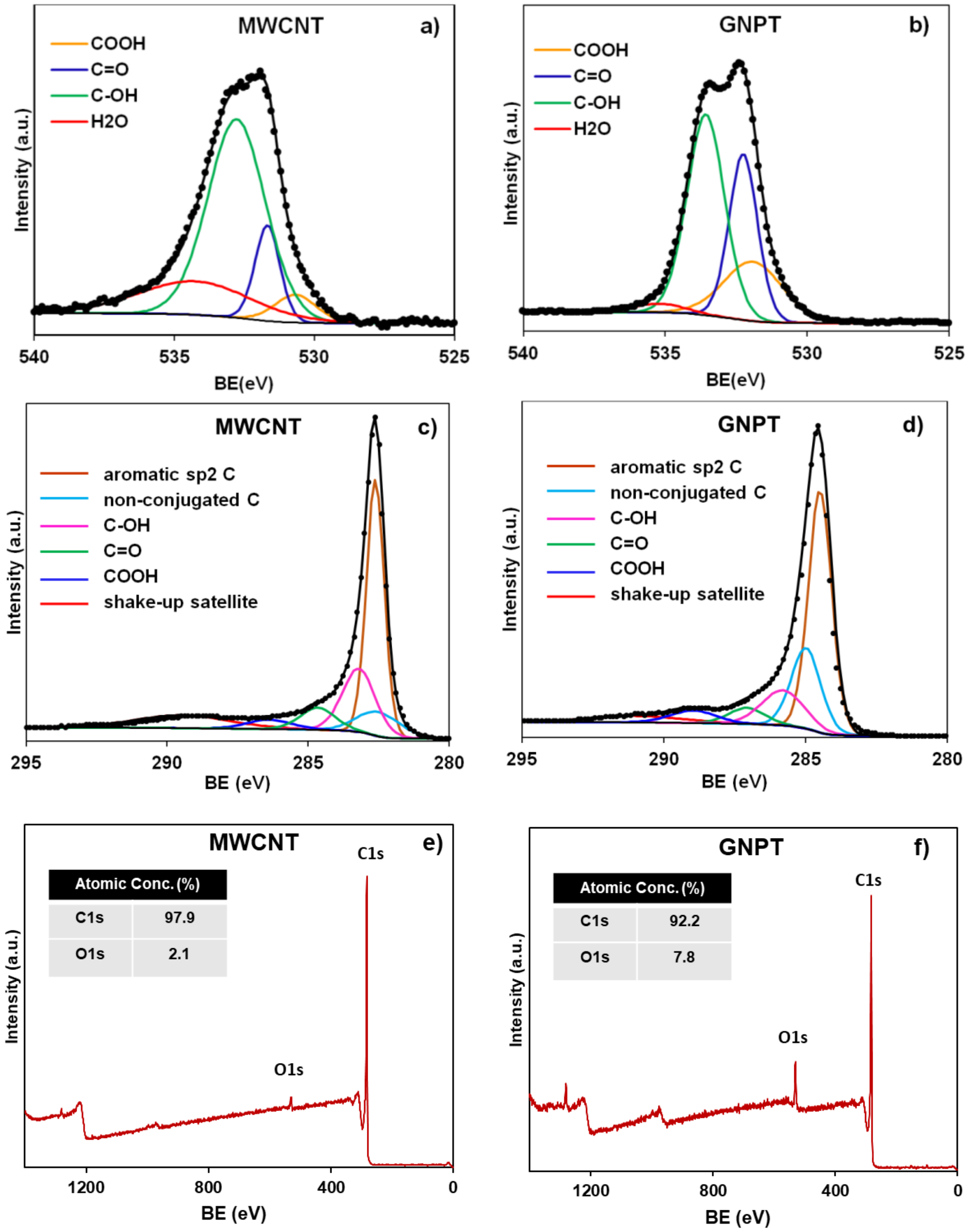

Figure S1. High resolution XPS spectra of MWCNT and GNPT in the O1s (a, b) and C1s regions (c, d), and survey XPS of MWCNT and GNPT with insets showing the chemical composition of the samples (e, f).

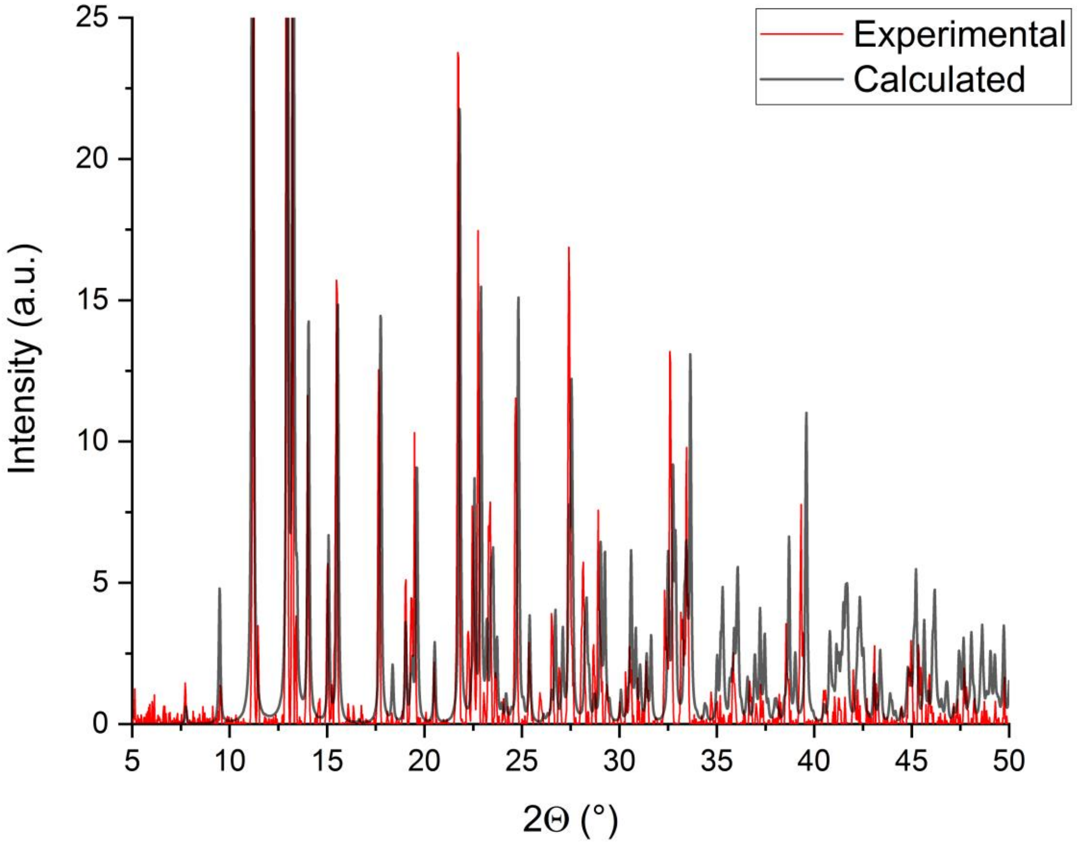

Figure S2. Experimental (red) and calculated (black) X-ray powder diffraction spectra for $[Pd(HL)Br_2]Cl_{0.74}Br_{0.26}\cdot H_2O$.

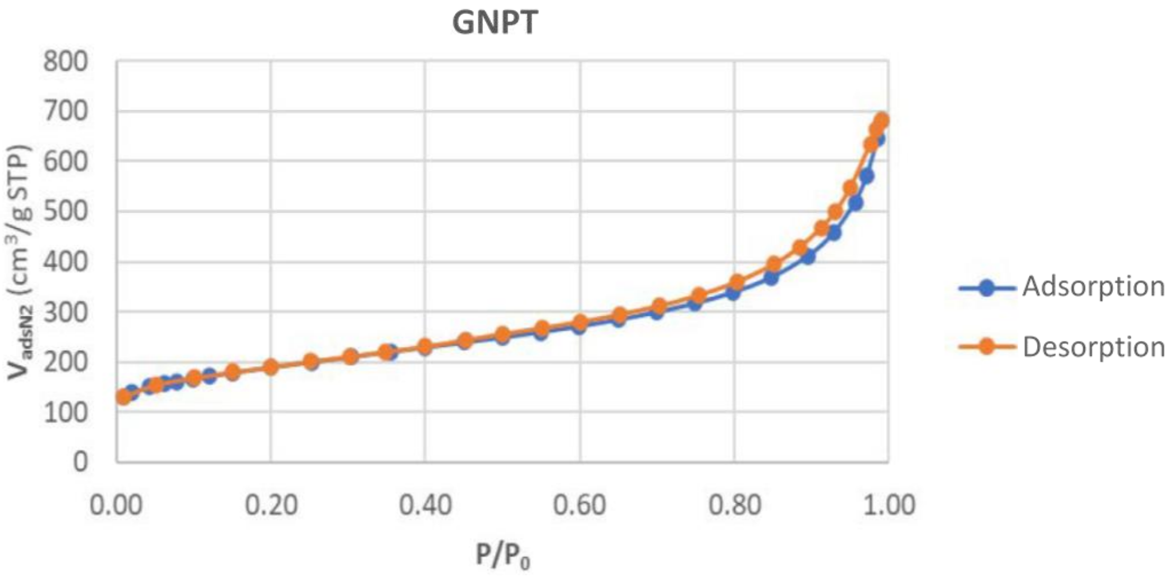

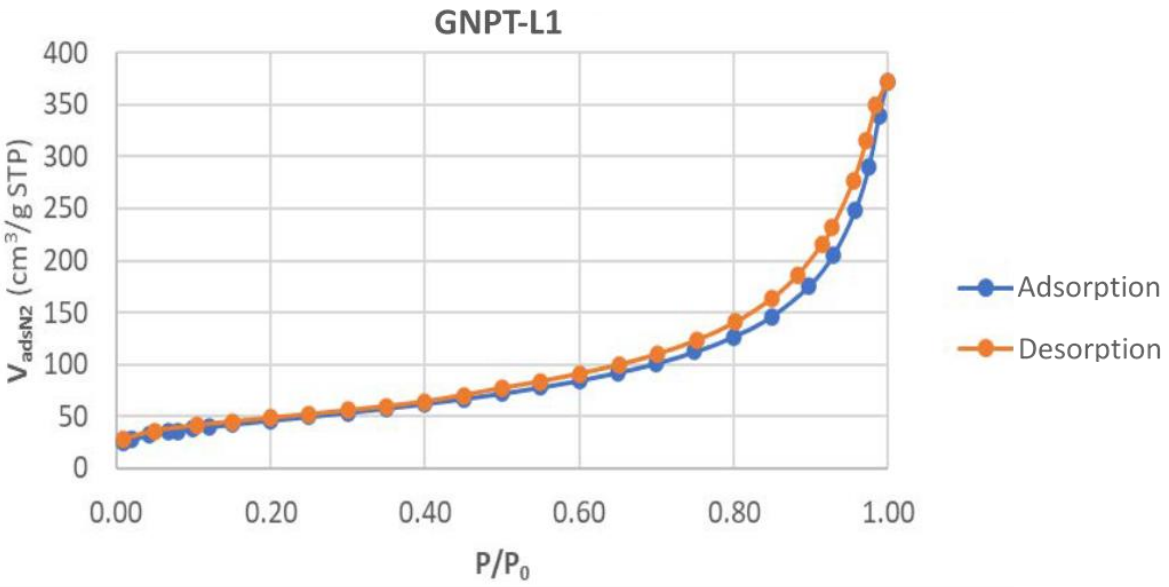

Figure S3. Nitrogen adsorption and desorption isotherms, at 77 K, of GNPT and GNPT-L1.

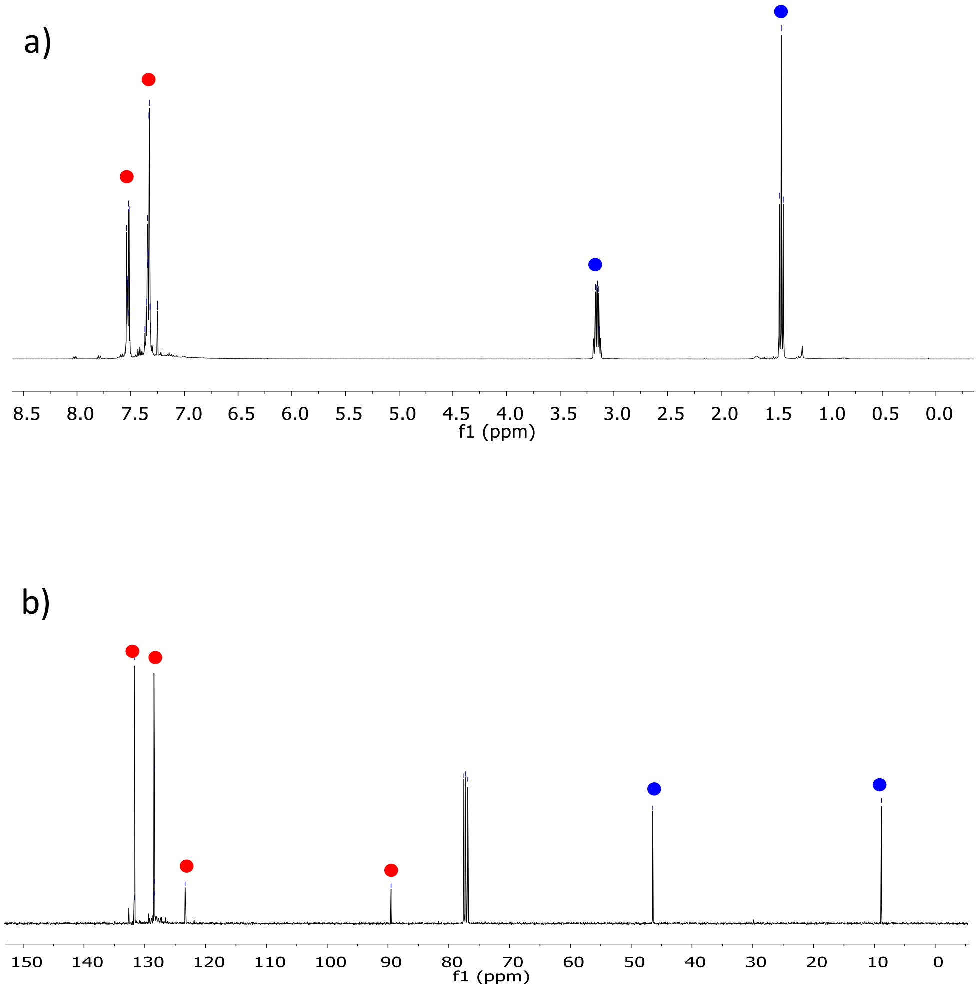

Figure S4. ^{1}H NMR (a) and ^{13}C NMR (b) spectra, in $CDCl_3$, of the crude product obtained from the reaction between IB and PA catalyzed by GNPT-L2-Pd (● signals assigned to DPA; ● signals assigned to Et_3N, see **Table S3**).

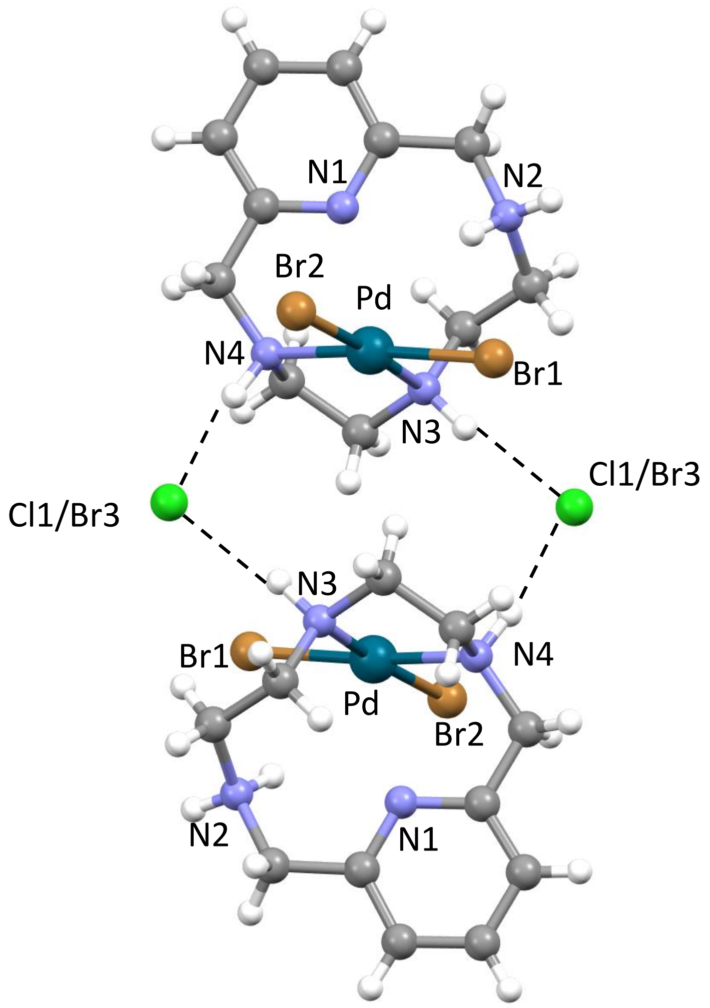

Figure S5. Centrosymmetric dimer of $[Pd(HL)Br_2]^+$ cations in $[Pd(HL)Br_2]Cl_{0.74}Br_{0.26}\cdot H_2O$, H-bonded to the bromide counterions.

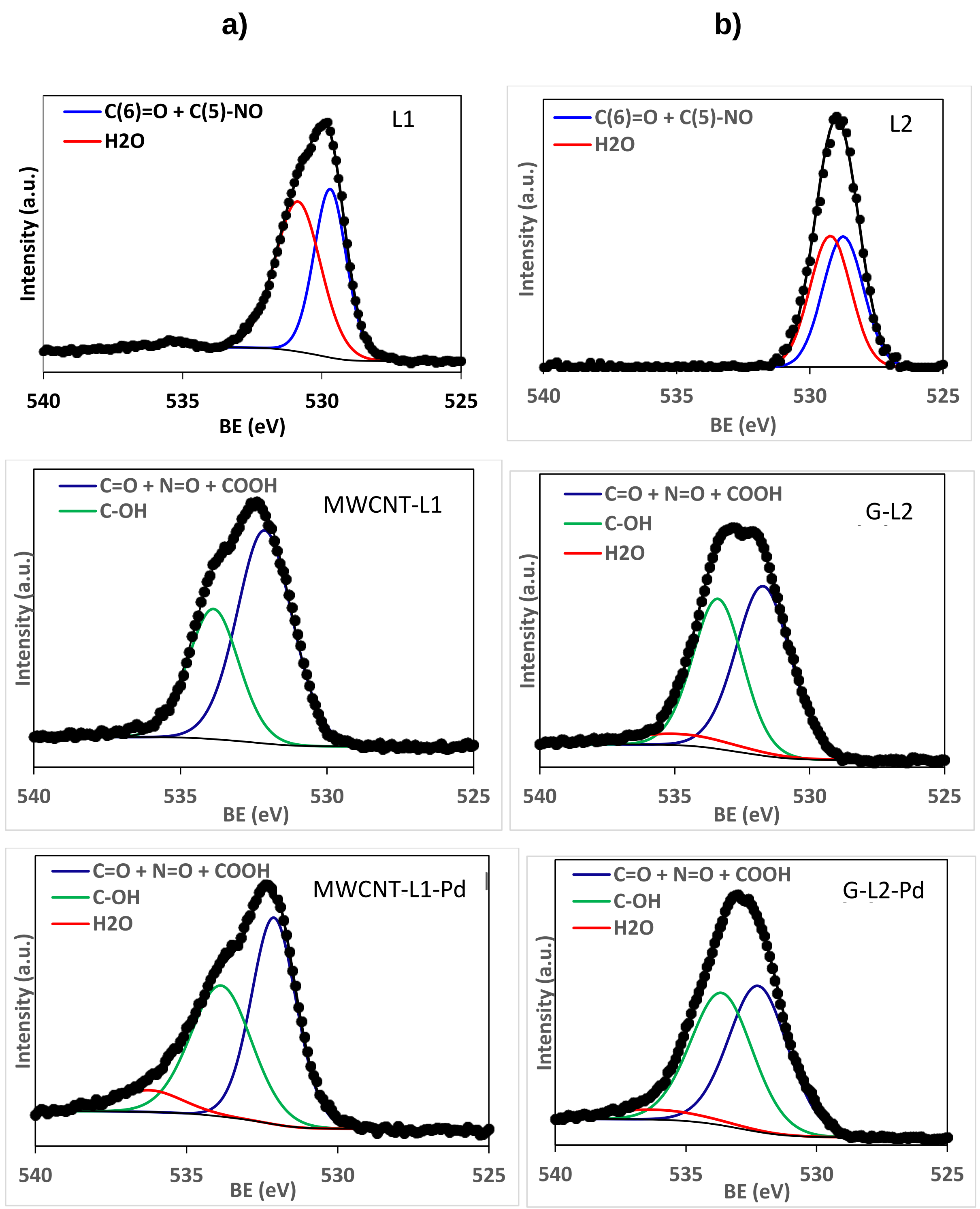

Figure S6. High-resolution XPS spectra in the O1s region of: a) L1, MWCNT-L1 and MWCNT-L1-Pd; b) L2, G-L2 and G-L2-Pd.

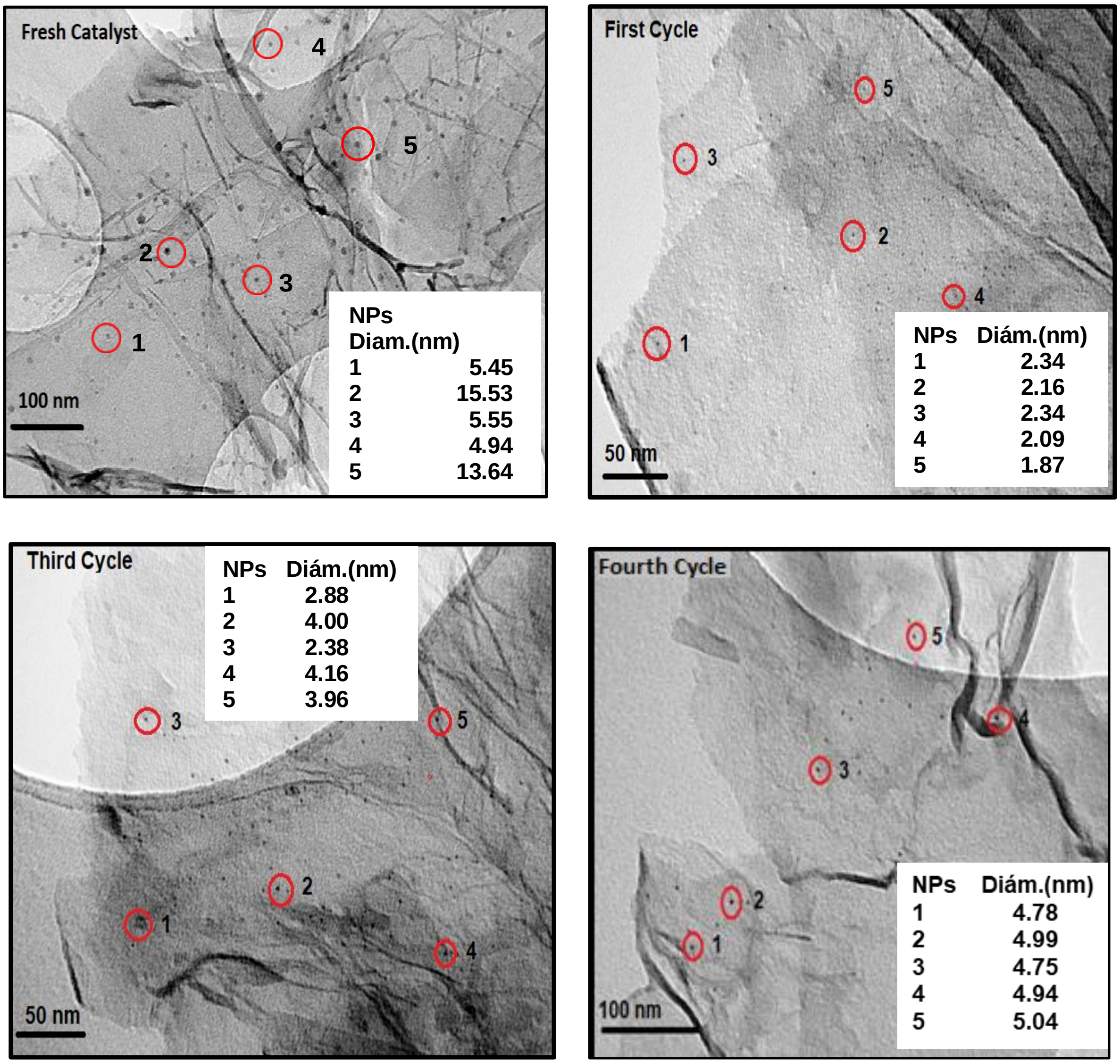

Figure S7. TEM micrographs of fresh and reused G-L2-Pd catalyst.

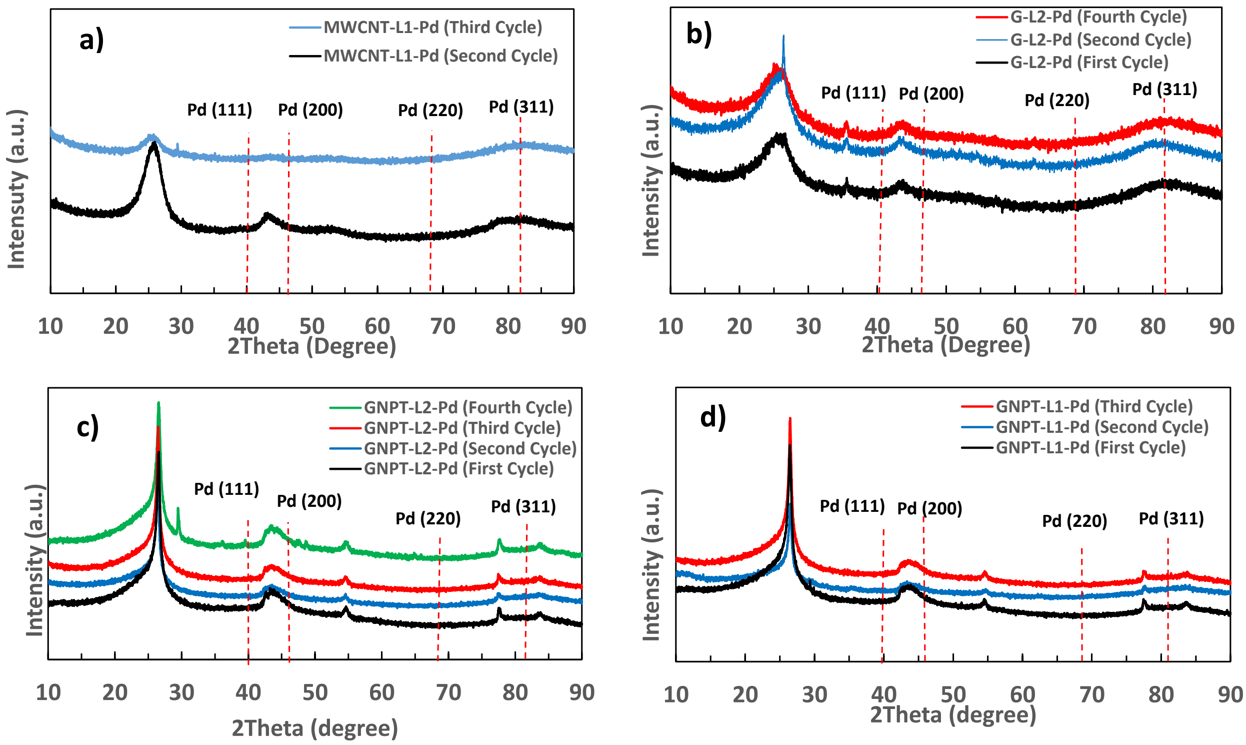

Figure S8. XRD diffraction patterns of: a) Cycles of MWCNT-L2-Pd; b) Cycles of G-L2-Pd; c) Cycles of GNPT-L2-Pd; d) Cycles GNPT-L1-Pd.

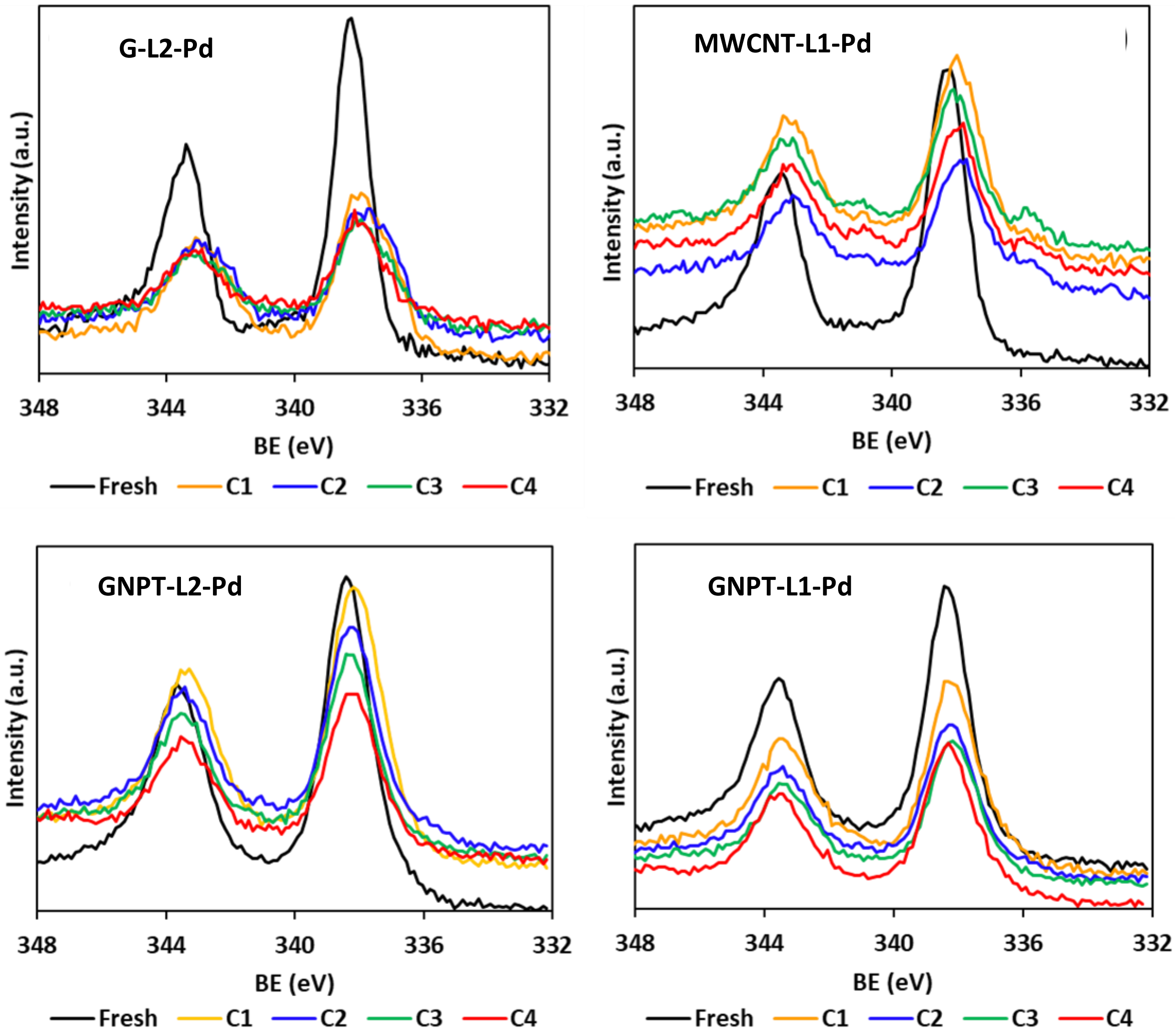

Figure S9. XPS spectra of fresh and reused (four cycles) catalysts in the Pd 3d region.

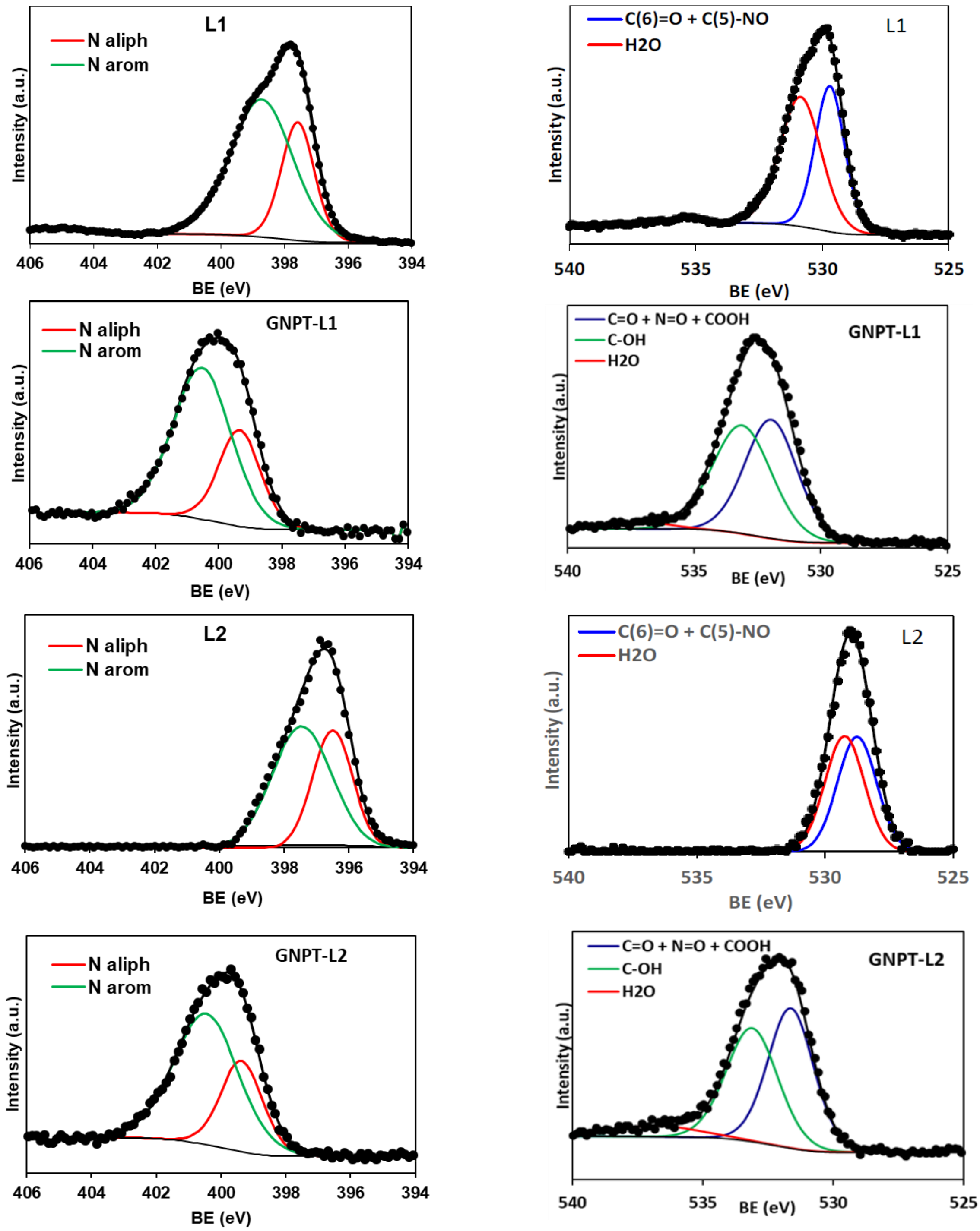

a) b)

Figure S10. High resolution XPS spectra of L1, GNPT-L1, L2 and GNPT-L2: a) in the N1s region, b) in the O1s region.

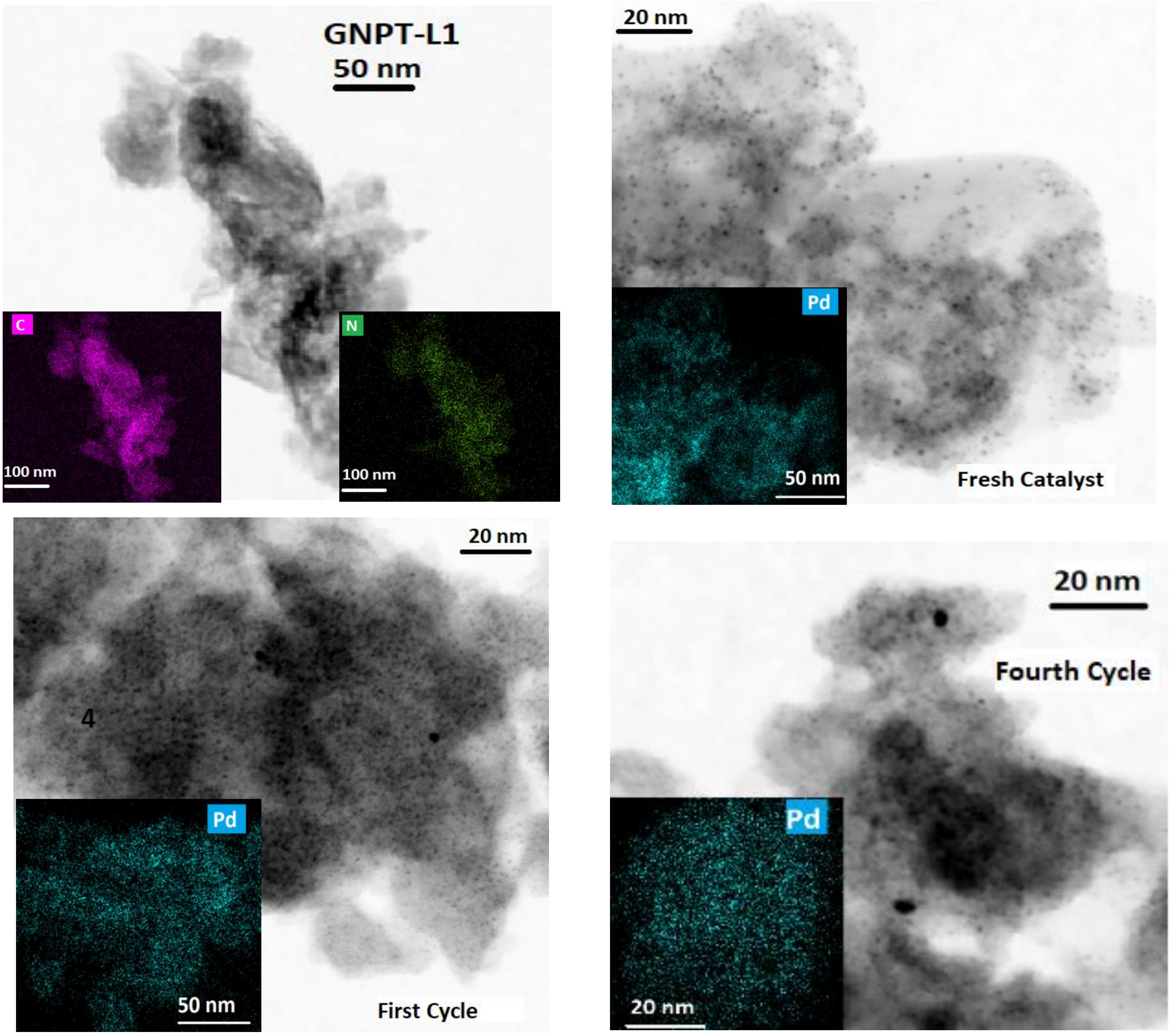

Figure S11. TEM micrographs of GNPT-L1-Pd: top) fresh catalyst, bottom left) catalyst after the first cycle, bottom right) catalyst after the fourth cycle. Insets: elements distribution maps.

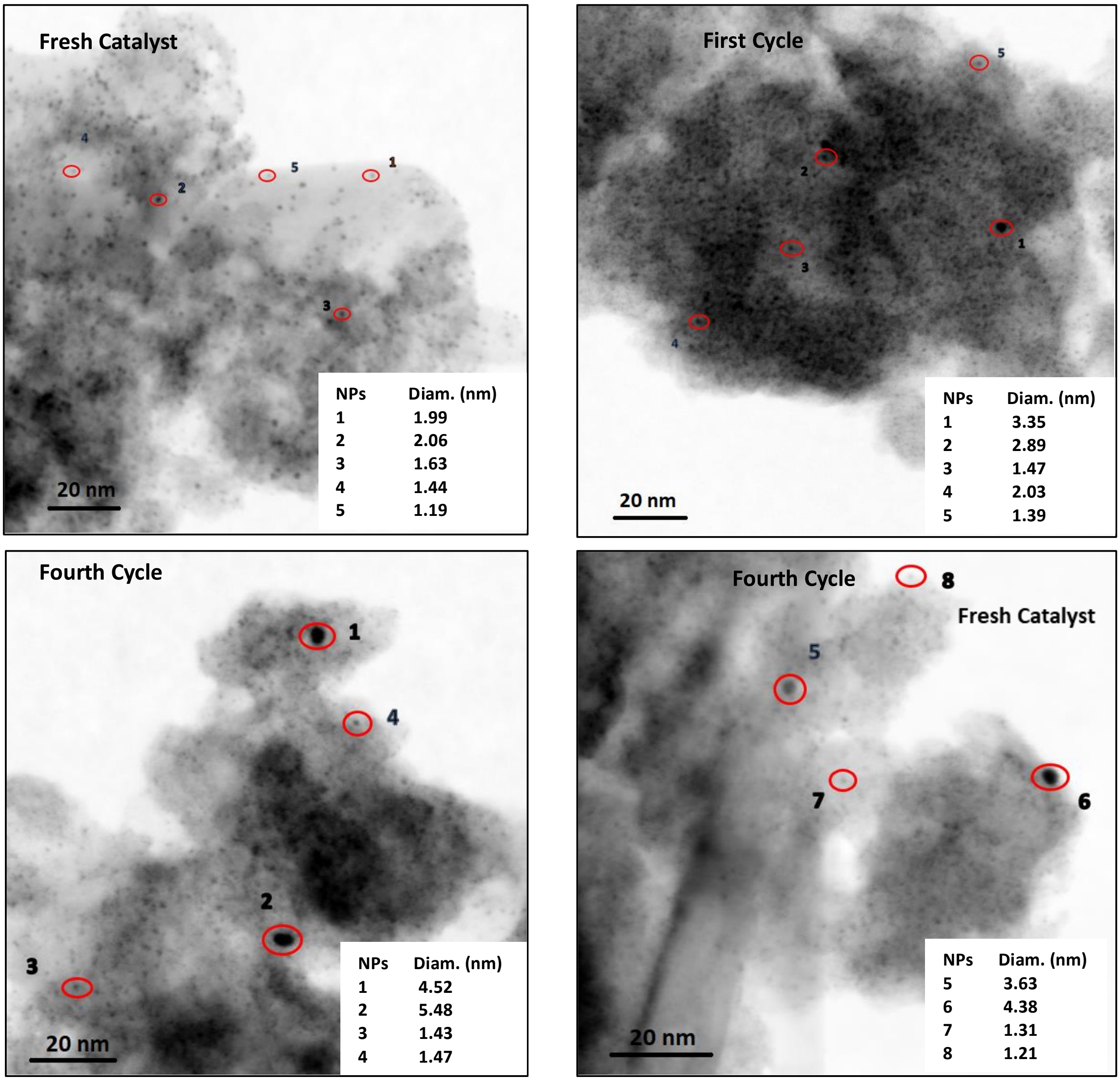

Figure S12. TEM micrographs of fresh and reused GNPT-L1-Pd catalyst.

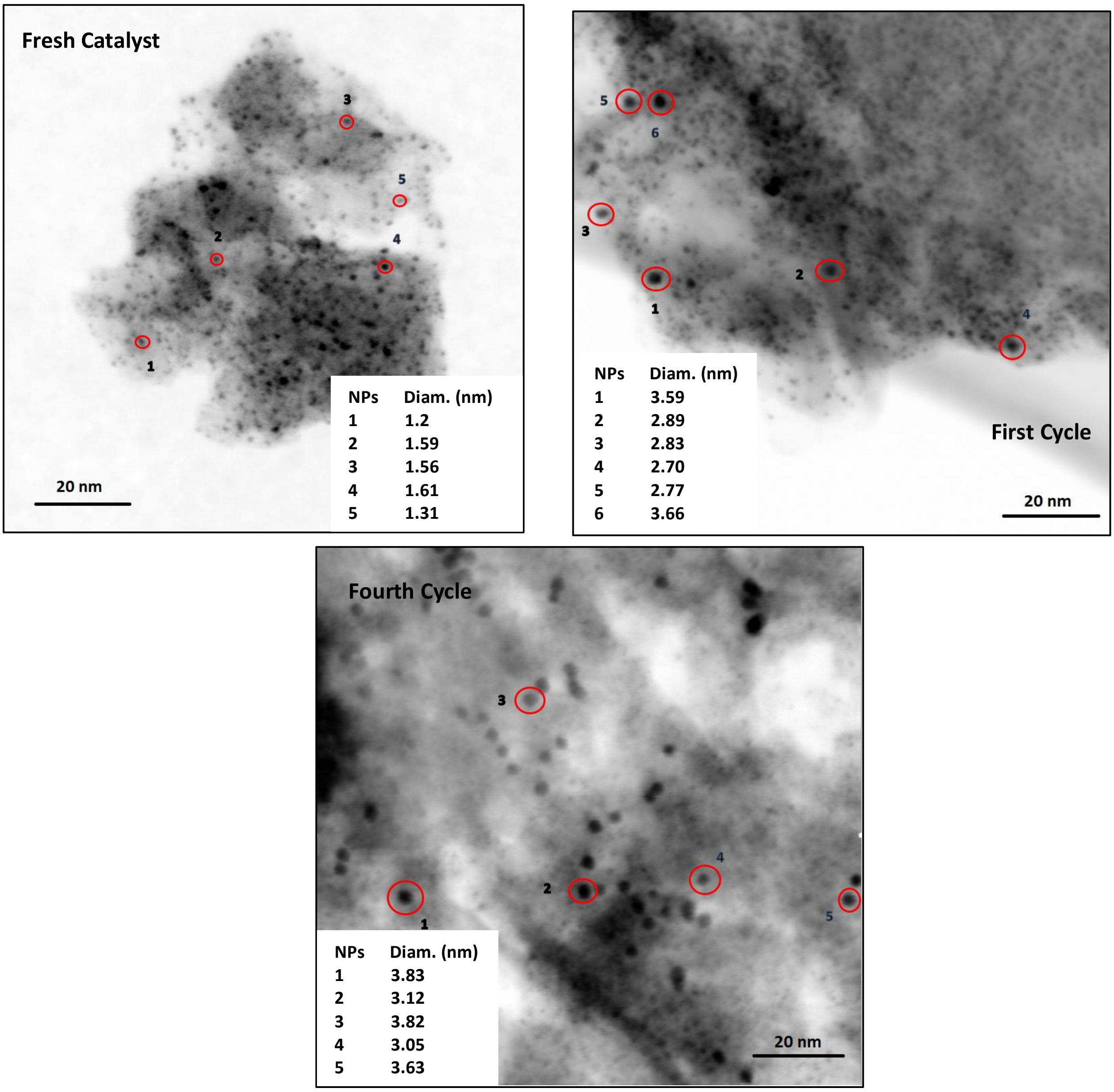

Figure S13. TEM micrographs of fresh and reused GNPT-L2-Pd catalyst.
